# Deep IDA: a deep learning approach for integrative discriminant analysis of multi-omics data with feature ranking—an application to COVID-19

**DOI:** 10.1093/bioadv/vbae060

**Published:** 2024-04-24

**Authors:** Jiuzhou Wang, Sandra E Safo

**Affiliations:** Division of Biostatistics and Health Data Science, University of Minnesota, Minneapolis, MN 55414, United States; Division of Biostatistics and Health Data Science, University of Minnesota, Minneapolis, MN 55414, United States

## Abstract

**Motivation:**

Many diseases are complex heterogeneous conditions that affect multiple organs in the body and depend on the interplay between several factors that include molecular and environmental factors, requiring a holistic approach to better understand disease pathobiology. Most existing methods for integrating data from multiple sources and classifying individuals into one of multiple classes or disease groups have mainly focused on linear relationships despite the complexity of these relationships. On the other hand, methods for nonlinear association and classification studies are limited in their ability to identify variables to aid in our understanding of the complexity of the disease or can be applied to only two data types.

**Results:**

We propose Deep Integrative Discriminant Analysis (IDA), a deep learning method to learn complex nonlinear transformations of two or more views such that resulting projections have maximum association and maximum separation. Further, we propose a feature ranking approach based on ensemble learning for interpretable results. We test Deep IDA on both simulated data and two large real-world datasets, including RNA sequencing, metabolomics, and proteomics data pertaining to COVID-19 severity. We identified signatures that better discriminated COVID-19 patient groups, and related to neurological conditions, cancer, and metabolic diseases, corroborating current research findings and heightening the need to study the post sequelae effects of COVID-19 to devise effective treatments and to improve patient care.

**Availability and implementation:**

Our algorithms are implemented in PyTorch and available at: https://github.com/JiuzhouW/DeepIDA

## 1 Introduction

Many diseases are complex heterogeneous conditions that affect multiple organs in the body and depend on the interplay between several factors that include molecular and environmental factors. Research studies continue to demonstrate that a better understanding of the complexity and heterogeneity in complex diseases requires analytical approach that goes beyond individual analysis of different types of data or views (e.g. genetics, proteomics, lipidomics). It is also widely recognized that deep learning methods are flexible in learning complex nonlinear relationships in data. However, most existing deep learning methods for integrative analysis are limited in their ability to produce clinically meaningful findings that shed light on the parthenogenesis of complex diseases. In this article, we develop a deep learning method for modeling complex nonlinear associations and separation in two or more data types while producing clinically meaningful results. In the literature, data from multiple sources are sometimes termed “views.” We use views, data types, and multiple sources interchangeably in this work.

### 1.1 Motivating application: a COVID-19 study

COVID-19 is due to complications from severe acute respiratory syndrome coronavirus 2 (SARS-CoV-2) but the clinical course of the infection varies for each individual. In particular, research suggests that patients with and without severe COVID-19 have different genetic, pathological, and clinical signatures (Severe-Covid-19-GWAS-Group 2020, Overmyer *et al.* 2021), highlighting the need to use multiple molecular data to better understand the disease. Our work is motivated by a study conducted by Overmyer *et al.* (2021) that used multi-omics data to understand COVID-19 mechanisms. Blood samples were collected from 128 patients admitted to Albany Medical Center, NY from 6 April 2020 to 1 May 2020 for moderate to severe respiratory problems. These samples were quantified for metabolomics, RNA sequencing (RNA-seq), proteomics, and lipidomics. In addition to molecular data, various demographic and clinical data were obtained at the time of enrollment. For eligibility, subjects had to be at least 18 years old and admitted to the hospital for COVID-19-like symptoms. Of the eligible, 102 had COVID-19 and 26 did not have COVID-19. Of those with COVID-19, 51 were admitted to the Intensive Care Unit (ICU) and 51 were not admitted to the ICU (i.e. Non-ICU). Of those without COVID-19, 10 were Non-ICU patients and 16 were ICU patients.

In Overmyer *et al.* (2021), the primary analyses involved separately associating each omics data with disease severity. Secondary analyses considered pairwise associations of the molecular data. Their findings suggest that the severity of COVID-19 could be related to dysregulation of the lipid transport system. In this article, we take a holistic approach to integrate molecular data and disease severity, defined in terms of COVID-19 status and ICU status. In particular, instead of assessing pairwise associations and using unsupervised statistical methods to correlate the different views, we aim to model the overall dependency structure among the views while simultaneously modeling separation between the COVID-19 patient groups. Ultimately, our goal is to elucidate the molecular architecture of COVID-19 by identifying molecular signatures that can discriminate between patients with and without COVID who were or were not admitted to the ICU.

### 1.2 Existing methods

Many linear methods have been proposed in the literature that could be used to associate molecular data. For example, canonical correlation analysis (CCA) methods have been proposed to learn linear projections of two views that are maximally correlated (Hotelling 1936, Carroll 1968, Safo *et al.* 2018). CCA with deep neural networks (DNN) (Deep CCA) (Andrew *et al.* 2013), and its variations (e.g. Wang *et al.* 2015, Benton *et al.* 2019), have been proposed to learn nonlinear projections of two or more views that are maximally correlated. Refer to Guo and Wu (2019) for a review of some CCA methods. These association-based methods are all unsupervised, and they do not use an outcome data (i.e. class labels) when learning the low-dimensional representations.

Methods have been proposed to learn linear projections of two or more views that simultaneously maximize association between views and separation between classes in each view (Safo *et al.* 2021, Zhang and Gaynanova 2021). For example, in Safo *et al.* (2021), a method that combined linear discriminant analysis (LDA) and CCA was proposed to maximize association of multiple views and separation of classes in each view. These methods have focused primarily on learning linear relationships. But the relationships among the multiple views and classes are oftentimes complex to be understood solely by linear methods. Nonlinear methods such as deep learning methods, could be used to model complex nonlinear structure among the views and between a view and the outcome.

Nonlinear methods have also been proposed to jointly multiview data and discriminate between classes (Hu *et al.* 2019, Gao *et al.* 2022, Moon and Lee 2022, Gao and Shang 2023). In Hu *et al.* (2019), a DNN method, named MvLDAN, was proposed to learn nonlinear projections of multiple views that maximally correlate the views and separate the classes in each view but the convergence of MvLDAN is not guaranteed. Importantly, MvLDAN and similar nonlinear association-based methods are not capable of ranking or selecting features, impacting interpretability and clinical applicability. For instance, applying MvLDAN to our motivating COVID-19 data would constrain our ability to identify key molecules driving the association between views and separation of the COVID-19 patient groups. Recently, a data integration and classification method (MOMA) for multiview learning that uses the attention mechanism for interpretability has been proposed (Moon and Lee 2022). Of note, the algorithm developed for MOMA is applicable to two views, which is very restrictive, and cannot be used for our motivating application with three views. In Mirzaei *et al.* (2019), a two-step approach for feature ranking that is based on a teacher-student (TS) framework was proposed. The “teacher” step obtains the best low-dimensional representation of the data using any dimension reduction method (e.g. Deep CCA). The “student” step performs feature ranking based on these low-dimensional representations. In particular, a single-layer network with sparse weights is trained to reconstruct the low-dimensional representations obtained from the “teacher” step, and the features are ranked based on the weights. The TS framework is limited because model training (i.e. identifying low-dimensional representations of the data) and feature ranking steps are separated, thus one cannot ensure that the top-ranked features identified are meaningful.

### 1.3 Our approach

Motivated by our goal of elucidating the molecular architecture of COVID-19 severity through the use of only few multi-omics features, we propose Deep Integrative Discriminant Analysis (IDA) to learn complex nonlinear relationships in these data. Our work makes several contributions in both methods and applications. First, the proposed method combines the flexibility of deep learning with advantages of CCA and LDA to simultaneously model complex nonlinear associations between two or more views and separations between classes in every view. Because we incorporate LDA in our framework for discrimination, we do not need any further sophisticated methods for classification such as support vector machine (SVM), which reduces the level of computational complexity. Second, since we want to identify molecular features that may shed light on the pathogenesis of COVID-19, we propose a feature ranking framework based on resampling techniques to identify features contributing most to the overall association of the views and the separation of the classes within a view. We emphasize that our feature ranking framework can be implemented in other classification problems. Third, we develop an algorithm that can handle high dimensional problems convergence guaranteed. Finally, our simulations and real data analyses demonstrate that the performance of the proposed approach is similar to or better than several existing deep learning methods even when the sample size is small relative to the number of variables. Supplementary Table S1 highlights the key features of Deep IDA.

The rest of the article is organized as follows. In Section 2, we introduce the proposed method and algorithms for implementing the method. In the Section 3, we use simulations to demonstrate the effectiveness of Deep IDA where there is “ground truth.” We also showcase the performance of Deep IDA on the motivating data, under small sample size setting. We include an additional application using MNIST handwriting data to primarily assess the classification performance of the proposed method without feature ranking, under large sample size setting. Application of our method to the motivating data will assess classification performance of Deep IDA and also demonstrate that Deep IDA can identify biologically relevant features. We end with a conclusion remark in Section 4. Due to space constraints, all proofs, optimization, linear simulations and parts of the real data analysis are given in the Supplementary material.

## 2 Methods

Let $X^{d}\in R^{n\times p_{d}}$ be the data matrix for view *d*, d=1,…,D (e.g. proeotimcs, metabolomics, RNA-seq, lipidomics data). Each view, $X^{d}$, has $p_{d}$ variables, all measured on the same set of *n* individuals or units. Suppose that each unit belongs to one of two or more classes, *K*. Let $y_{i},i=1,\ldots ,n$ be the class membership for unit *i*. For each view, let $X^{d}$ be a concatenation of data from each class, i.e. $X^{d}={\left[X_{1}^{d},X_{2}^{d},\ldots ,X_{K}^{d}\right]}^{T}$, where $X_{k}^{d}\in R^{n_{k}\times p_{d}},k=1,\ldots ,K$ and $n=\sum_{k=1}^{K}n_{k}$. For the *k*-th class in the *d*-th view, $X_{k}^{d}={\left[x_{k,1}^{d},x_{k,2}^{d},\ldots ,x_{k,n_{k}}^{d}\right]}^{T}$, where $x_{k,i}^{d}\in R^{p_{d}}$ is a column vector denoting the view *d* data values for the *i*-th unit in the *k*-th class. Given the views and data on class membership, we wish to explore the association among the views and the separation of the classes simultaneously, and also to predict the class membership of a new unit using the unit’s data from all views or from some of the views. Additionally, we wish to identify features that contribute most to the overall association among the views and the separation of classes within each view. Recently, Safo *et al.* (2021) proposed sparse SIDA, a joint method that combines the integration step with the separation step. They showed that this joint method often leads to better classification accuracy compared to two-step methods: association followed by classification. We briefly describe this method in Supplementary material as it is relevant to the principles we propose here.

### 2.1 Deep integrative discriminant analysis

**Figure 1.**
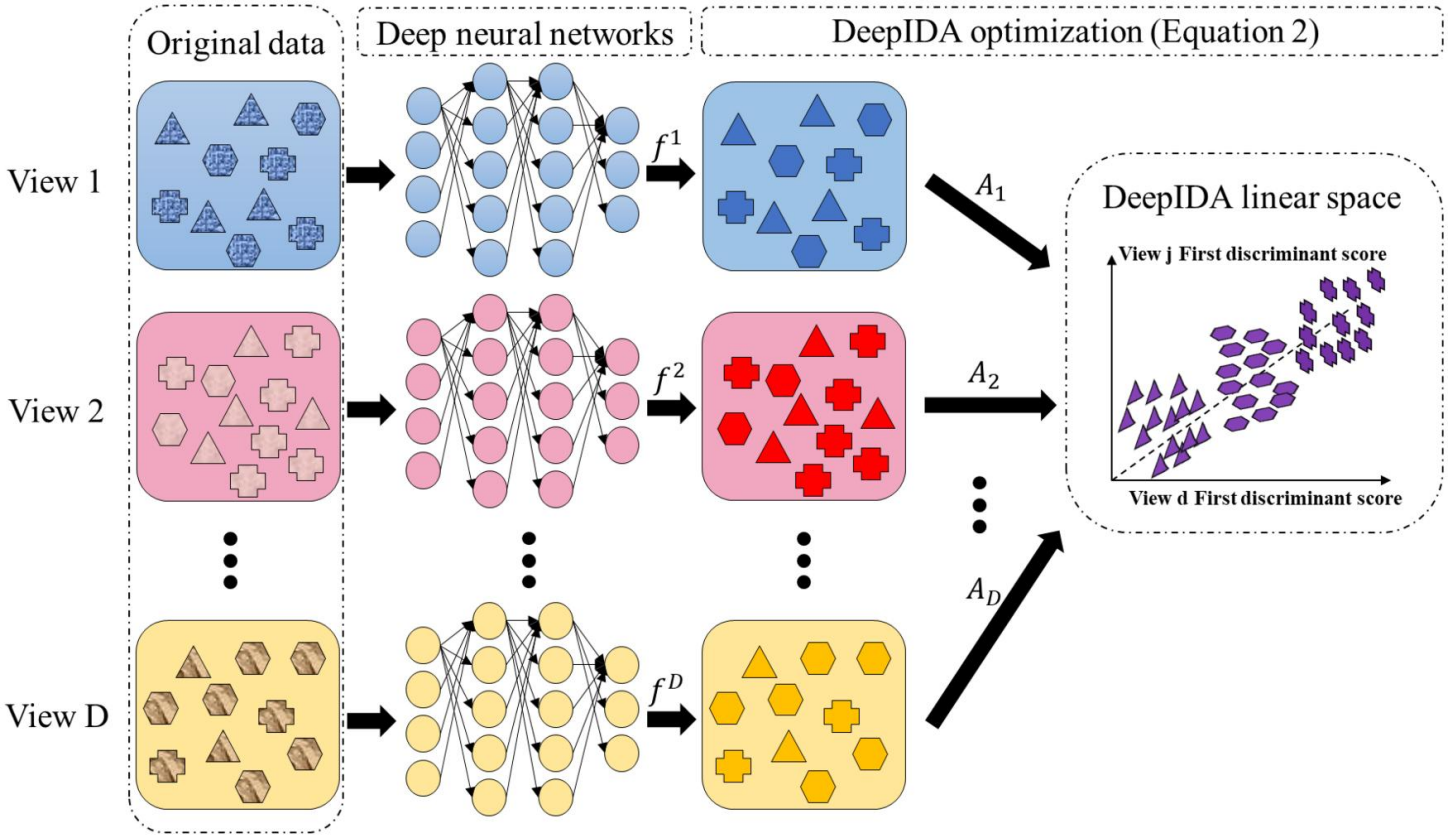
The framework of Deep IDA. Classes are represented by shapes and views are represented by colors. The (DNN are used to learn nonlinear transformations of the *D* views, the outputs of the DNN for the views ($f^{d}$) are used as inputs in the optimization problem, and we learn linear projections $A_{d},d=1,\ldots ,D$ that maximally correlate the nonlinearly transformed views and separate the classes within each view.

We extend the joint association and classification linear method in Safo *et al.* (2021) to learn nonlinear relationships between two or more views and between a view and a binary or multiclass outcome. We follow notations in Andrew *et al.* (2013) to define our deep learning network. Assume that the DNN has m=1,…,M layers for view *d* (each view can have its own number of layers), and each layer has $c_{m}^{d}$ nodes, for m=1,…,M − 1. Let $o_{1},o_{2},\ldots ,o_{D}$ be the dimensions of the final(*M*th) layer for the *D* views. Let $h_{1}^{d}=s(W_{1}^{d}x^{d}\;+\;b_{1}^{d})\;\in R^{c_{1}^{d}}$ be the output of the first layer for view *d*. Here, $x^{d}$ is a length-$p^{d}$ vector representing a row in $X^{d}$, $W_{1}^{d}\in R^{c_{1}^{d}\times p^{d}}$ is a matrix of weights for view *d*, $b_{1}^{d}\in R^{c_{1}^{d}}$ is a vector of biases for view *d* in the first layer, and s∈R→R is a nonlinear activation function. Using $h_{1}^{d}$ as an input for the second layer, let the output of the second layer be denoted as $h_{2}^{d}=s(W_{2}^{d}h_{1}^{d}\;+\;b_{2}^{d})\in R^{c_{2}^{d}}$, $W_{2}^{d}\in R^{c_{2}^{d}\times c_{1}^{d}}$ and $b_{2}^{d}\in R^{c_{2}^{d}}$. Continuing in this fashion, let the output of the (m−1)th layer be $h_{m-1}^{d}=s(W_{m-1}^{d}h_{m-2}^{d}\;+\;b_{m-1}^{d})\in R^{c_{m-1}^{d}}$, $W_{m-1}^{d}\in R^{c_{m-1}^{d}\times c_{m-2}^{d}}$ and $b_{m-1}^{d}\in R^{c_{m-1}^{d}}$. Denote the output of the final layer as $f^{d}(x^{d},\theta^{d})=s(W_{M}^{d}h_{M-1}^{d}\;+\;b_{M}^{d})\in R^{o_{d}}$, where $\theta^{d}$ is a collection of all weights, $W_{m}^{d}$, and biases, $b_{m}^{d}$ for m=1,…,M and d=1,…,D. In matrix notation, the output of the final layer of the *d*-th view is denoted as $H^{d}=f^{d}(X^{d})\in R^{n\times o_{d}}$, where it is clear that $f^{d}$ depends on the network parameters. On this final layer, we propose to solve a modified IDA optimization problem to obtain projection matrices that maximally associate the views and separate the classes. Specifically, we propose to find a set of linear transformations $A_{d}=[\alpha_{d,1},\alpha_{d,2},\ldots ,\alpha_{d,l}]\in R^{o_{d}\times l}$, $l\;\leq \;\min\{K-1,o_{1},\ldots ,o_{D}\}$ such that when the nonlinearly transformed data are projected onto these linear spaces, the views will have maximum linear association and the classes within each view will be maximally linearly separated. Figure 1 is a visual representation of Deep IDA.

For a specific view *d*, $H^{d}={\left[H_{1}^{d},H_{2}^{d},\ldots ,H_{K}^{d}\right]}^{T},H_{k}^{d}\in R^{n_{k}\times o_{d}},k=1,\ldots ,K$ and $n=\sum_{k=1}^{K}n_{k}$. For the *k*-th class in the *d*-th final output, $H_{k}^{d}={\left[h_{k,1}^{d},h_{k,2}^{d},\ldots ,h_{k,n_{k}}^{d}\right]}^{T}$, where $h_{k,i}^{d}\in R^{o_{d}}$ is a column vector representing the output for subject *i* in the *k*th class for view *d*. Using $H^{d}$, the output of the final layer, as the data matrix for view *d*, we define the between-class covariance (i.e. $S_{b}^{d}\in R^{o_{d}\times o_{d}}$), the total covariance (i.e. $S_{t}^{d}\in R^{o_{d}\times o_{d}}$), and the cross-covariance between view *d* and *j* ($S_{dj}\in R^{o_{d}\times o_{j}}$), respectively, as: $S_{b}^{d}=\frac{1}{n-1}\sum_{k=1}^{K}n_{k}(\mu_{k}^{d}-\mu^{d}){\left(\mu_{k}^{d}-\mu^{d}\right)}^{T}$; $S_{t}^{d}=\frac{1}{n-1}\sum_{i=1}^{n}(h_{k,i}^{d}-\mu^{d}){\left(h_{k,i}^{d}-\mu^{d}\right)}^{T}=\frac{1}{n-1}(H^{d^{T}}-\mu^{d}\cdot 1){\left(H^{d^{T}}-\mu^{d}\cdot 1\right)}^{T}$, and $S_{dj}=\frac{1}{n-1}(H^{d^{T}}-\mu^{d}\cdot 1){\left(H^{j^{T}}-\mu^{j}\cdot 1\right)}^{T}$. Here, 1 is an all-ones row vector of dimension *n*, $\mu_{k}^{d}=\frac{1}{n_{k}}\sum_{i=1}^{n_{k}}h_{k,i}^{d}\in R^{o_{d}}$ is the *k*-th class mean, and $\mu^{d}=\frac{1}{K}\sum_{i=1}^{K}\mu_{k}^{d}\in R^{o_{d}}$ is the mean for projected view *d*.

To obtain the linear transformations $A_{1},A_{2},\ldots ,A_{D}$ and the parameters of Deep IDA defining the functions $f^{d}$, (i.e. the weights and biases), we propose to solve the following:

(1) $$\underset{A_{1},\ldots ,A_{D},f^{1},\ldots ,f^{D}}{\arg\max}\{\frac{\rho }{D}\sum_{d=1}^{D}tr[A_{d}^{T}S_{b}^{d}A_{d}]+\frac{2(1-\rho )}{D(D-1)}\sum_{d=1}^{D}\sum_{j,j\neq d}^{D}tr[A_{d}^{T}S_{dj}A_{j}A_{j}^{T}S_{dj}^{T}A_{d}]\}\;\text{subject}\;\text{to}\;\text{tr}[A_{d}^{T}S_{t}^{d}A_{d}]=l,\forall d.$$

where tr[] is the trace of a matrix and ρ is a hyper-parameter that controls the relative contribution of the separation and the association to the optimization problem. Through extensive simulations, we have found that a ρ value of 0.5 yields good classification performance. However, users can perform cross-validation to find the best ρ. Here, the first term is an average of the separation for the *D* views, and the second term is an average of pairwise squared correlations between two different views ($\frac{D(D-1)}{2}$ measures in total).

For fixed Deep IDA parameters, (i.e. weights and biases), (1) reduces to solving the optimization problem:

(2) $$\underset{A_{1},\ldots ,A_{D}}{\arg\max}\{\frac{\rho }{D}\sum_{d=1}^{D}tr[A_{d}^{T}S_{b}^{d}A_{d}]+\frac{2(1-\rho )}{D(D-1)}\sum_{d=1}^{D}\sum_{j,j\neq d}^{D}tr[A_{d}^{T}S_{dj}A_{j}A_{j}^{T}S_{dj}^{T}A_{d}]\}\;\text{subject}\;\text{to}\;\text{tr}[A_{d}^{T}S_{t}^{d}A_{d}]=l,\forall d.$$

Denote $S_{t}^{d}{\;}^{-\frac{1}{2}}$ as the square root of the inverse of $S_{t}^{d}$. With the assumption that $o_{d}<n$, $S_{t}^{d}$ is non-singular, as such we can take the inverse. Let $M^{d}=S_{t}^{d}{\;}^{-\frac{1}{2}}S_{b}^{d}S_{t}^{d}{\;}^{-\frac{1}{2}}$, $N_{dj}=S_{t}^{d}{\;}^{-\frac{1}{2}}S_{dj}S_{t}^{j}{\;}^{-\frac{1}{2}}$ and $\Gamma_{d}=S_{t}^{d}{\;}^{\frac{1}{2}}A_{d}$. Then, the optimization problem in (2) is equivalently

(3) $$\underset{\Gamma_{1},\Gamma_{2},\ldots ,\Gamma_{D}}{\arg\max}\{\frac{\rho }{D}\sum_{d=1}^{D}tr[\Gamma_{d}^{T}M^{d}\Gamma_{d}]+\frac{2(1-\rho )}{D(D-1)}\sum_{d=1}^{D}\sum_{j,j\neq d}^{D}tr[\Gamma_{d}^{T}N_{dj}\Gamma_{j}\Gamma_{j}^{T}N_{dj}^{T}\Gamma_{d}]\}\;\text{subject}\;\text{to}\;\text{tr}[\Gamma_{d}^{T}\Gamma_{d}]=l,\forall d.$$

and the solution reduces to solving a system of eigenvalue problems. More formally, we have the following theorem.

Theorem 1 Let $S_{t}^{d}$ and $S_{b}^{d}$ respectively be the total covariance and the between-class covariance for the top-level representations $H^{d},d=1,\ldots ,D$. Let $S_{dj}$ be the cross-covariance between top-level representations *d* and *j*. Assume $S_{t}^{d}≻0$. Define $M^{d}=S_{t}^{d}{\;}^{-\frac{1}{2}}S_{b}^{d}S_{t}^{d}{\;}^{-\frac{1}{2}}$ and $N_{dj}=S_{t}^{d}{\;}^{-\frac{1}{2}}S_{dj}S_{t}^{j}{\;}^{-\frac{1}{2}}$. Then $\Gamma^{d}\in R^{o_{d}\times l}$, $l\leq \min\{K-1,o_{1},\ldots ,o_{D}\}$ in (3) are eigenvectors corresponding respectively to eigenvalues $\Lambda_{d}=$diag$\left(\lambda_{d_{k}},\ldots ,\lambda_{d_{l}}\right)$, $\lambda_{d_{k}}>\cdots >\lambda_{d_{l}}>0$ that iteratively solve the eigensystem problems: $\left(c_{1}M^{d}+c_{2}\sum_{j\neq d}^{D}N_{dj}\Gamma_{j}\Gamma_{j}^{T}N_{dj}^{T}\right)\Gamma_{d}=\Lambda_{d}\Gamma_{d},\forall d=1,\ldots ,D,$ where $c_{1}=\frac{\rho }{D}$ and $c_{2}=\frac{2(1-\rho )}{D(D-1)}$.

The proof of Theorem 1 is in the Supplementary material. We can initialize the algorithm using any arbitrary normalized nonzero matrix. After iteratively solving *D* eigensystem problems until convergence, we obtain the optimized linear transformations ${\tilde{\Gamma }}_{1},\ldots ,{\tilde{\Gamma }}_{D}$ which maximize both separation of classes in the top-level representations, $H^{d}$, and the association among the top-level representations. Since we find the eigenvector-eigenvalue pairs of $\left(c_{1}M^{d}+c_{2}\sum_{j=1,j\neq d}^{D}N_{dj}\Gamma_{j}\Gamma_{j}^{T}N_{dj}^{T}\right)$, the columns of ${\tilde{\Gamma }}_{d}$, d=1,…,D are orthogonal and provide unique information that contributes to the association and separation in the top-level representations. Given the optimized linear transformations ${\tilde{\Gamma }}_{1},\ldots ,{\tilde{\Gamma }}_{D}$, we construct the objective function for the *D* DNNs as:

(4) $$\underset{f^{1},f^{2},\ldots ,f^{D}}{\arg\max}\{c_{1}\sum_{d=1}^{D}tr[{\tilde{\Gamma }}_{d}^{T}M^{d}{\tilde{\Gamma }}_{d}]+c_{2}\sum_{d=1}^{D}\sum_{j,j\neq d}^{D}tr[{\tilde{\Gamma }}_{d}^{T}N_{dj}{\tilde{\Gamma }}_{j}{\tilde{\Gamma }}_{j}^{T}N_{dj}^{T}{\tilde{\Gamma }}_{d}]\}.$$

Theorem 2 For *d* fixed, let $\eta_{d,1},\ldots ,\eta_{d,l}$, $l\leq \min\{K-1,o_{1},\ldots ,o_{D}\}$ be the largest *l* eigenvalues of: $c_{1}M^{d}+c_{2}\sum_{j\neq d}^{D}N_{dj}\Gamma_{j}\Gamma_{j}^{T}N_{dj}^{T}$. Then the solution ${\tilde{f}}^{d}$ to the optimization problem in (4) for view d maximizes: $\sum_{r=1}^{l}\eta_{d,r}$.

The proof of Theorem 2 is in the Supplementary material. The objective function in Theorem 2 aims to maximize the sum of the *l* largest eigenvalues for each view. In obtaining the view-specific eigenvalues, we use the cross-covariances between that view and each of the other views, and the total and between-class covariances for that view. Thus, by maximizing the sum of the eigenvalues, we are estimating corresponding eigenvectors that maximize both the association of the views and the separation of the classes within each view. By Theorem 2, the solution ${\tilde{f}}^{1},\ldots ,{\tilde{f}}^{D}$, i.e. weights and biases for the *D* neural networks of the optimization problem (4) is also given by the following:

(5) $$\underset{f^{1},f^{2},\ldots ,f^{D}}{\arg\max}\sum_{d=1}^{D}\sum_{r=1}^{l}\eta_{d,r}.$$

The objectives (3) and (5) are naturally bounded because the characteristic roots of every $S_{t}^{d}{\;}^{-1}S_{b}^{d}$ (and hence $S_{t}^{d}{\;}^{-\frac{1}{2}}S_{b}^{d}S_{t}^{d}{\;}^{-\frac{1}{2}}$) is bounded and every squared correlation is also bounded. This guarantees convergent solutions of the loss function in (5) compared to the method in (Dorfer *et al.* 2015) that constrain the within-group covariance and has unbounded loss function. We optimize the objective in (5) with respect to the weights and biases for each layer and each view to obtain $\tilde{f^{1}},\ldots ,\tilde{f^{D}}$. The estimates $\tilde{f^{1}}(X^{1}),\ldots ,\tilde{f^{D}}(X^{D})$ are used as the low-dimensional representation to classify events.

Of note, since Deep IDA already implements a discriminant analysis method when obtaining the low-dimensional representations that separate the classes in a view, we do not need any sophisticated classification methods (such as SVM which tends to be computationally expensive for large sample sizes) when predicting future events. Thus, for classifying future events, we follow the approach in Safo *et al.* (2021) and we use nearest centroid to assign events to the class with the closest mean. For this purpose, we have the option to use the pooled low-dimensional representations $\hat{f}=(\tilde{f^{1}}(X^{1}),\ldots ,\tilde{f^{D}}(X^{D}))$ or the individual estimates $\tilde{f^{d}}(X^{d}),d=1,\ldots ,D$. If testing data are available, say $X_{\text{test}}^{d}$, we use the learned neural network parameters to construct the output of the top-level representations for each view, i.e. $H_{\text{test}}^{d}=\tilde{f^{d}}(X_{\text{test}}^{d}),d=1,\ldots ,D$. These are then used in a nearest centroid classifier (NCC) to predict the test classes. We emphasize that the process of nonlinearly transforming the original data using DNNs, estimating parameters for each neural network, and obtaining linear projections that jointly maximize association of the views and separation of the classes is what we call Deep IDA. We implement the proposed algorithm as a Python 3.0 package with dependencies on NumPy (Oliphant 2006) and PyTorch Paszke *et al.* (2019) for numerical computations, and Matlab for model visualization. For the network’s depth, we recommend 8 layers for datasets with both sample and feature sizes larger than 1000, and 3 layers for simpler scenarios where the sample dimension is around 100. Cross-validation can be used to select the structure with best performance while avoiding overfitting. We include a thorough comparison of our proposed method with existing methods in Supplementary Section S1.3. Details of the optimization process and proposed algorithm are discussed in Supplementary Section S1.3.

### 2.2 Feature ranking via Bi-Bootstrap sampling

A main limitation of most existing nonlinear methods for integrating data from multiple views is that it is difficult to interpret the models and this limits their ability to produce clinically meaningful findings. This is especially important for our motivating application. We propose a general framework for ranking features in deep learning models for data from multiple views that is based on ensemble learning. We refer to our sampling approach as *Bi-Bootstrap Sampling*, since we get bootstrap samples for both sample index and feature index. We implement Deep IDA on different bootstrap datasets to yield baseline classification accuracy, we permute the data and use this data and the Deep IDA learned model to investigate and rank variables based on how often a variable result in a decrease in classification accuracy when permuted. One could also obtain low-dimensional representations of the data based on the top-ranked variables, which would require another Deep IDA training.

**Figure 2.**
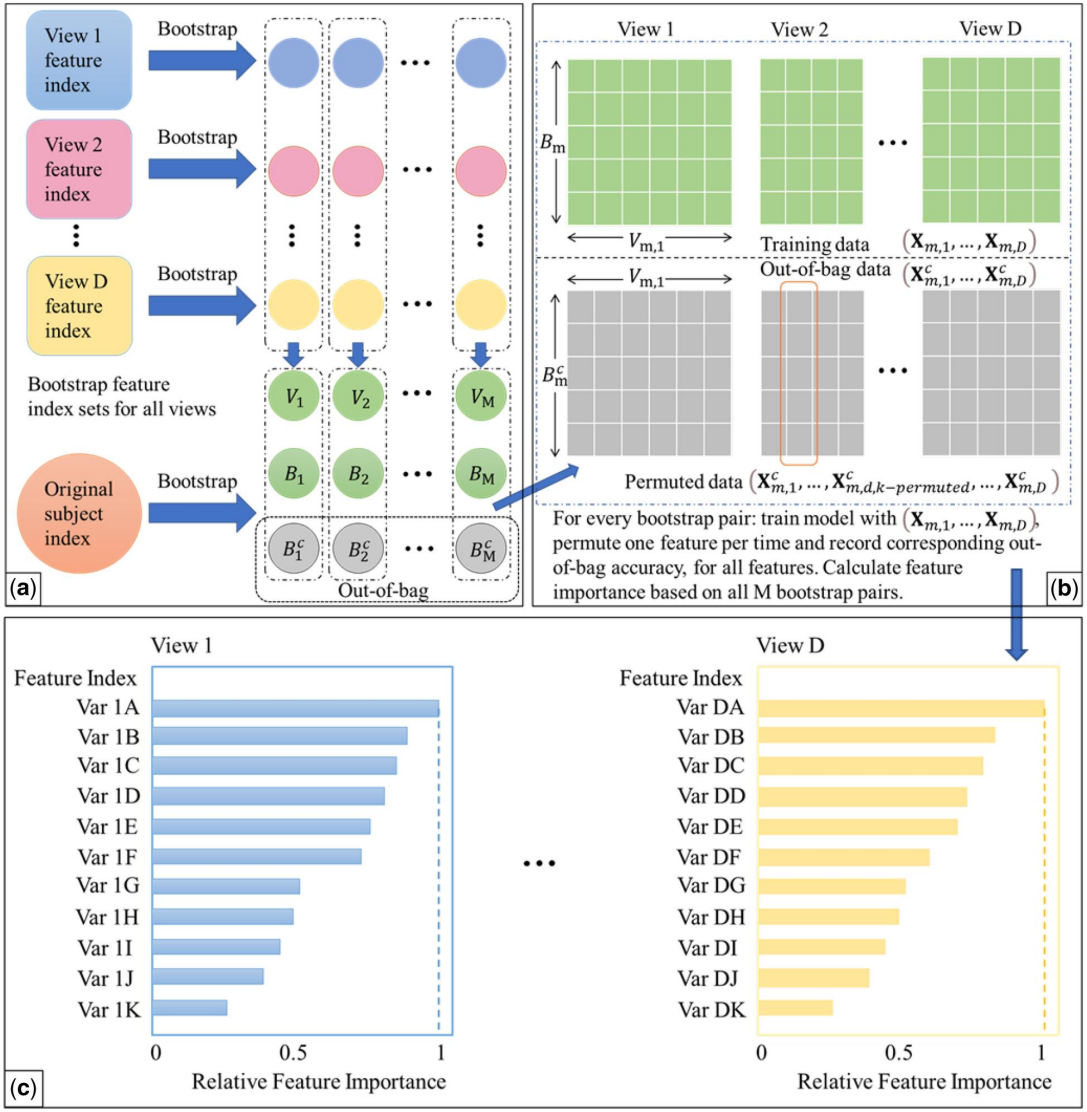
The framework of feature ranking process. (a) Bootstrapping samples and features. It includes Steps 1 and 2. $V_{m}$: the *m*-th bootstrap feature index; $B_{m}$: the *m*-th bootstrap sample index; $B_{m}^{c}$: the *m*-th bootstrap out-of-bag sample index. (b) Pairing data, training the reference model, permuting and recording the decrease in classification performance. This includes Steps 3–6. (c) Ranking features based on how often the baseline classification accuracy is reduced when permuted. This includes Steps 7 and 8.

We emphasize that while we embed Deep IDA in this feature ranking procedure, in principle, any method for associating multiple views can be embedded in this process. This makes the proposed approach general. We outline our feature ranking steps below. Figure 2 is a visual representation of the feature ranking procedure.

1. Generate *M* bootstrap sets of sample indices of the same sample size as the original data by random sampling the indices with replacement. Denote the bootstrap sets of indices as $B_{1},B_{2},\ldots ,B_{M}$. Let the out-of-bag sets of indices be $B_{1}^{c},B_{2}^{c},\ldots ,B_{M}^{c}$. In generating the bootstrap training sets of indices, we use stratified random sampling to ensure that the proportions of samples in each class in bootstrap sets of indices are similar to original data.
2. Draw *q* bootstrap sets of feature indices for each view. For view *j*, j=1,…,D, draw $0.8p_{j}$ samples from the index set and denote as $V_{m,j}$. $V_{m}=\{V_{m,1},V_{m,2},\ldots ,V_{m,D}\}$ is the *m*-th bootstrap feature index for all *D* views.
3. Pair sample and feature index sets randomly and denote as $\left(B_{1},V_{1}),\ldots ,(B_{M},V_{M}\right)$. For each pair $\left(B_{m},V_{m}\right)$ and $\left(B_{m}^{c},V_{m}\right)$ extract corresponding subsets of data.
4. For the *m*th pair, denote the bootstrap data as $X_{m,1},\ldots ,X_{m,D}$ and the out-of-bag data as $X_{m,1}^{c},\ldots ,X_{m,D}^{c}$. Train Deep IDA based on $X_{m,1},\ldots ,X_{m,D}$, and calculate the test classification rate based on $X_{m,1}^{c},\ldots ,X_{m,D}^{c}$. Record this rate as baseline classification rate for pair m,m=1,2,…,M.
5. For the *d*th view in the *m*th pair, permute the *k*th variable in $X_{m,d}^{c}$ and keep all other variables unchanged. Denote the permuted view *d* data as $X_{m,d,k-\text{permuted}}^{c}$. Use the learned model from Step 4 and the permuted data $\left(X_{m,1}^{c},\ldots ,X_{m,d,k-\text{permuted}}^{c},\ldots ,X_{m,D}^{c}\right)$ to obtain the classification rate for the permuted data.
6. Repeat Step 5 for m=1,…,M, d=1,…,D, and $k=1,\ldots ,p_{d}$. Record the variables that lead to a decrease in classification rate when using the permuted data.
7. For the *d*th view, calculate the occurrence proportion of variable *k*, $k=1,2,\ldots ,p_{d}$ (i.e. the proportion of times a variable leads to a decrease in classification accuracy) as $\frac{n_{k}}{N_{k}}$, where $n_{k}$ denotes the number of times that permuting variable *k* leads to a decrease in out-of-bag classification rate, and $N_{k}$ denotes the number of times that variable *k* is permuted (i.e. the total number of times variable *k* is selected in the bootstrap feature index sets). Repeat this process for all views.
8. For each view, rank the variables based on the occurrence proportions and select the top-ranked variables as the important variables. The top-ranked variables could be the top *r* variables or top r% variables.
9. (Optional) Once we have obtained the top-ranked variables for each view, we train Deep IDA on the original data but with these top-ranked variables. This step will yield classification accuracy based on just the top-ranked features, if that is desired. The number of variables needs to be specified in advance by domain knowledge and 10 % is used by default. To determine the optimal number of top features, one can employ cross-validation, starting with the top 1% of features and incrementally adding more until model accuracy is no longer significantly improved.

The Bi-Bootstrap process provides insights into how each variable in each view contributes to the classification performance of our nonlinear model. For each bootstrap sample, we only need to train Deep IDA once, and then every out-of-bag sample with one permuted variable is used in the learned Deep IDA model to assess classification performance. We note that the permutation step is easily parallelizable for computational efficiency. The top-ranked variables can also be used in other classification methods to enhance classification accuracy. Furthermore, the feature ranking procedure is model agnostic and intuitive, so step (4) can easily be substituted with other classification methods besides Deep IDA. We can replace the classification rate with other metrics, such as balanced accuracy or weighted *F*1 score, for imbalanced dataset.

Our optimization algorithm and run time analysis are documented in the Supplementary material.

## 3 Results

### 3.1 Simulation studies

#### 3.1.1 Set-up

We conduct simulation studies to assess the performance of Deep IDA for varying data dimensions, and as the relationship between the views and within a view become more complex. We demonstrate that Deep IDA is capable of (i) simultaneous association of data from multiple views and discrimination of sample classes and (ii) identifying signal variables and eliminating noise variables.

We consider two different simulation Scenarios. In Scenario One, we simulate data to have linear relationships between views and linear decision boundaries between classes (refer to Supplementary material for set-up and results). In Scenario Two, we simulate data to have nonlinear relationships between views and nonlinear decision boundaries between classes. In Scenario One, we explore two different settings: one with D=2 views and another with D=3 views, across K=3 classes. In Scenario Two, there are K=2 classes and D=2 views. In all Scenarios, we generate 20 Monte Carlo training, validation, and testing sets. We evaluate the proposed and existing methods using the following criteria: (i) classification performance [via test accuracy, area under the operating characteristic curve (AUROC), precision, recall, and *F*1 measure] and (ii) feature selection performance [via true positive rate (TPR), false positive rate (FPR), and *F*1]. For feature selection, we evaluate the methods ability to select the true signals (TPRs) while ignoring noise variables (FPRs). In Scenario One, the first 20 variables are important, and in Scenario Two, the Top 10% of variables in view 1 are signals. We compare test accuracy for Deep IDA with and without the variable ranking approach proposed in this manuscript.

#### 3.1.2 Comparison methods

Deep IDA is compared with association- and classification-only methods, allowing us to investigate the advantage of a joint association and classification method. For association-based methods, we consider the following methods: deep canonical correlation analysis (Deep CCA) (Andrew *et al.* 2013), sparse CCA via penalized-multivariate analysis (PMA)[Witten *et al.* (2009), sparse CCA via SELP (Safo *et al.* 2018), and deep generalized CCA (DGCCA) (Benton *et al.* 2019) when there are three or more views. The association-based methods are purely unsupervised, therefore, for classification, we use the learned low-dimensional representations from these methods as inputs in logistic regression models. For classification-only method, we consider SVM (Hastie *et al.* 2009) applied on stacked views, allowing us to investigate the benefits of data integration. We had wanted to include Deep LDA (Dorfer *et al.* 2015) in our analysis to assess the effectiveness of a classification-only nonlinear method compared to Deep IDA, a joint integration and classification method. However, due to convergence challenges caused by its unbounded loss function, we could not obtain valid estimates for a reliable comparison. Therefore, these results are excluded from our analyses. In implementing the comparison methods, we used Pytorch, for Deep CCA and Deep GCCA, and R packages mvlearnR (Palzer and Safo 2024) and PMA (Witten *et al.* 2013), for SELP and PMA, respectively.

To evaluate the impact of feature selection on classification performance, we first assessed the feature selection accuracy of our Bi-Bootstrap + Deep IDA framework against existing methods. This involves coupling Deep IDA and Deep CCA with the TS framework (Mirzaei *et al.* 2019) for variable ranking, and comparing with penalized methods like PMA and Sparse CCA via SELP. Similar to the linear setting, the top 10% ranked variables are selected for Bi-bootstrap and TS. We then implement Deep IDA, Deep CCA, and SVM on the training data but with just the Bi-Bootstrap selected variables. Notably, since PMA and SELP inherently include feature selection, they are trained using the complete feature set.

All methods are tested on both views, with additional results for View 1 only, as it encompasses all significant features. We experiment with various numbers of layers and nodes for each simulation scenario and choose the optimal setup for Deep IDA and Deep CCA. Details of the network structures are provided in the Supplementary material. All SVM analyses utilize linear kernels, maintaining parity with the assumed linearization of the low-rank representations in Deep IDA and Deep CCA after DNN processing.

#### 3.1.3 Nonlinear simulations

##### 3.1.3.1 Simulation design

**Figure 3.**
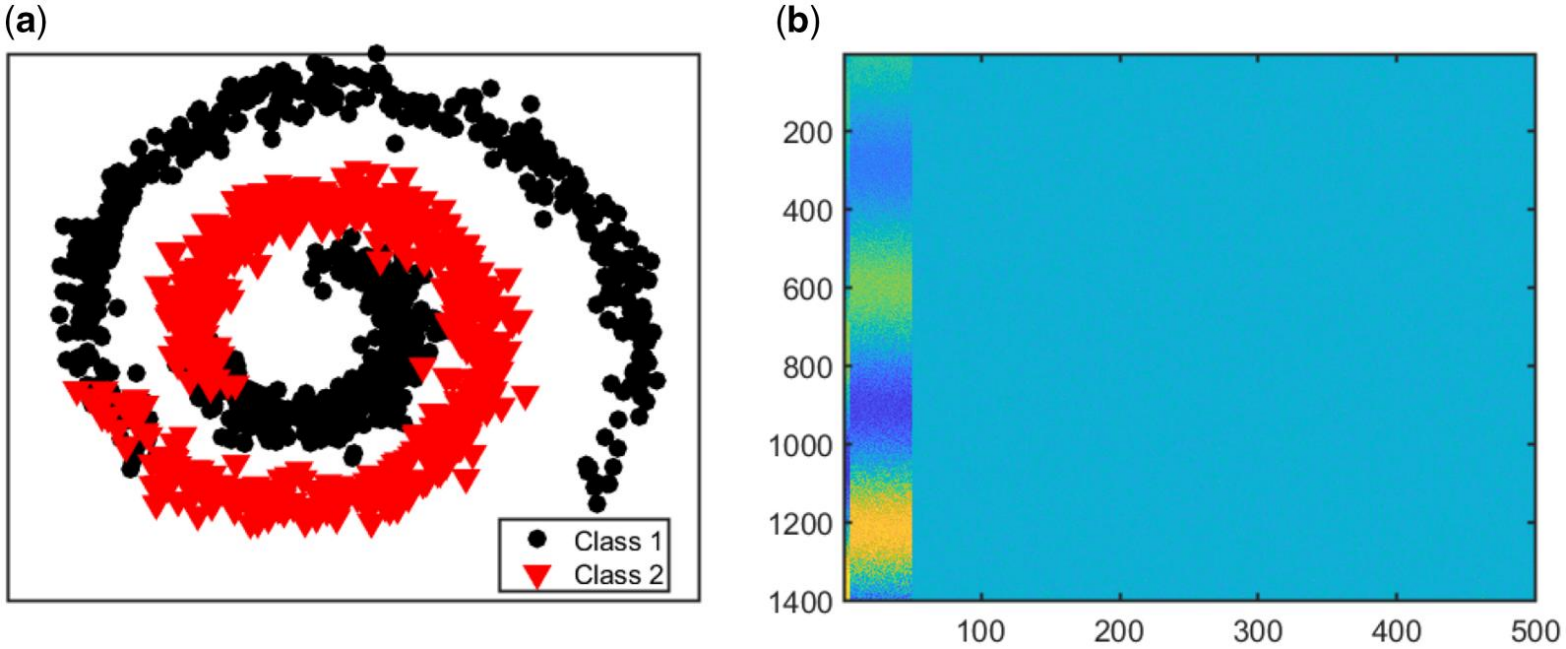
Setting one. (a) Structure of nonlinear relationships between signal variables in view 1. (b) Image plot of view 1 showing the first 50 variables as signals.

Details of linear simulations are in the Supplementary material. In our nonlinear simulations, we explore four distinct settings to evaluate the performance of each method under various combinations of sample and feature sizes. Let $n_{1}$ and $n_{2}$ represent the number of samples in each class, with the total sample size being $n=n_{1}+n_{2}$. The data in View 1 and View 2 are represented as $X_{1}:n\times p_{1}$ and $X_{2}:n\times p_{2}$, respectively. The four settings are defined as follows. S1: both number of features and samples are small, $n_{1}=200,n_{2}=150,p_{1}=p_{2}=500$; S2: large sample size, small feature size, $n_{1}=3000,n_{2}=2250,p_{1}=p_{2}=500$; S3: small sample size, large feature size, $n_{1}=200,n_{2}=150,p_{1}=p_{2}=2000$; S4: large sample and feature size, $n_{1}=3000,n_{2}=2250,p_{1}=p_{2}=2000$. In each setting, 10% of the variables in the first view are signals and the first five signal variables in the first view are related to the remaining signal variables in a nonlinear way (See Fig. 3). We generate data for View 1 as: $X_{1}={\tilde{X}}_{1}\cdot W+0.2E_{1}$ where (·) is element-wise multiplication, $W\in R^{n\times p_{1}}=[1_{0.1\times p_{1}},0_{0.9\times p_{1}}]$ is a matrix of ones and zeros, 1 is a matrix of ones, 0 is matrix of zeros, and $E_{1}∼N(0,1)$. The first five columns (or variables) of ${\tilde{X}}_{1}\in R^{n\times p^{1}}$ are simulated from exp(0.15θ)· sin(1.5θ). The next $0.1p^{1}-5$ variables are simulated from exp(0.15θ)· cos(1.5θ). Here, $\theta =\tilde{\theta }+0.5U(0,1)$, and $\tilde{\theta }$ is a vector of *n* evenly spaced points between 0 and 3π. The remaining $0.9p^{1}$ variables (or columns) in ${\tilde{X}}_{1}$ are generated from the standard normal distribution. View 2 has no signal variables and the variables do not have nonlinear relationships. Data for View 2 are generated as follows. We set each variable in $X_{1}$ with negative entries to zero, normalized each variable to have unit norm and added a random number generated from U(0,1).

##### 3.1.3.2 Simulation results

Table 1 gives variable selection accuracy for the methods compared. Bi-Bootstrap has similar TPRs when compared to SELP, except for Setting 4 where SELP is slightly better. However, the variation in the true positives for SELP is generally higher. PMA achieves slightly higher TPRs than our approach in all settings except setting 4 but it suffers from high false positives since the optimal loadings are mostly not sparse. TS framework hardly selects important variables. Our approach consistently selects most of the relevant variables with low false positives and negatives. Consequently, we examine whether this accurate variable selection translates into improved classification accuracy when methods are trained only on the selected variables by our proposed Bi-bootstrap.

**Table 1.** Comparison of feature selection performances across four scenarios.

Method	TPR	FPR	*F*1
**Setting1**			
Bi-Bootstrap + Deep IDA	1.00(0.00)	0.00(0.00)	1.00(0.00)
TS + Deep CCA	0.08(0.03)	0.10(0.01)	0.08(0.03)
Sparse CCA via SELP	0.87(0.14)	0.00(0.00)	0.92(0.11)
PMA	1.00(0.00)	1.00(0.00)	0.18(0.00)
**Setting2**			
Bi-Bootstrap + Deep IDA	0.64(0.06)	0.04(0.01)	0.64(0.06)
TS + Deep CCA	0.10(0.03)	0.10(0.00)	0.08(0.03)
Sparse CCA via SELP	0.87(0.13)	0.00(0.00)	0.92(0.10)
PMA	1.00(0.00)	1.00(0.00)	0.18(0.00)
**Setting3**			
Bi-Bootstrap + Deep IDA	0.96(0.02)	0.00(0.00)	0.96(0.02)
TS + Deep CCA	0.09 (0.02)	0.10(0.00)	0.09(0.02)
Sparse CCA via SELP	0.98(0.00)	0.00(0.00)	0.99(0.00)
PMA	0.84(0.29)	0.73(0.44)	0.27(0.18)
**Setting4**			
Bi-Bootstrap + Deep IDA	0.83(0.04)	0.02(0.01)	0.83(0.04)
TS + Deep CCA	0.14(0.06)	0.10(0.01)	0.14(0.06)
Sparse CCA via SELP	0.93(0.21)	0.00(0.00)	0.94(0.00)
PMA	1.00(0.00)	1.00(0.00)	0.18(0.00)

This table presents the TPR, FPR, and *F*1 Score for various feature selection methods across four scenarios in nonlinear simulations. Each entry displays the mean values derived from 20 simulations, with the standard deviation provided in parentheses. All values rounded to 2 digits.

Table 2 gives the classification metrics. Since in this simulation setup, only view 1 had informative features, we expected the classification accuracy from view 1 to be better than that from both views and this is what we observed across most methods. The classification accuracy, the area under the ROC curve (AUROC), precision, recall and *F*1 scores of Deep IDA was generally higher than the other methods, except in Setting Three where its precision was lower than Deep CCA on the whole data. We compared the classification metrics of the proposed method with Deep IDA on top selected features and without feature ranking by Bi-bootstrap (i.e. Deep IDA) to assess the effect variable selection has on classification estimates from our deep learning models. The improvement is the most significant in Setting 3, where the sample size is much lower than the feature size so feature selection can boost up the performance, where in other settings the performances are similar. Deep IDA on top selected features has competitive or better classification accuracy (especially when using view 1 only for classification) compared to all other non-Deep IDA based methods. Further, the classification metrics for Deep IDA on top selected variables or not is generally higher than the other methods applied to data with variables selected by Deep IDA + Bootstrap (e.g. Deep CCA on top 50 selected features, Setting One). The methods with built-in feature selection (PMA and sparse CCA) perform worse than Deep IDA as well. SVM applied on both views stacked together and on just view 1, either using the whole data or using data with variables selected by Deep IDA, resulted in similar classification performance, albeit lower than the proposed method. Thus, in this example, although only view 1 had signal variables, the classification performance from using both views in training was generally better than using only view 1 (e.g. SVM on view 1), attesting to the benefit of multiview analyses.

**Table 2.** Performance metrics for nonlinear simulations across four scenarios.

	**Setting1** (*n*1 = 200, *n*2 = 150, *p*1 = 500, *p*2 = 500)					**Setting2** (*n*1 = 3000, *n*2 = 2250, *p*1 = 500, *p*2 = 500)				
Method	Accuracy	AUROC	*F*1	Precision	Recall	Accuracy	AUROC	*F*1	Precision	Recall
Deep IDA on top 50 selected features view 1	0.82(0.19)	0.81(0.21)	0.86(0.15)	0.85(0.18)	**0.90**(0.15)	0.93(0.07)	0.93(0.07)	**0.94**(0.06)	0.93(0.07)	**0.96**(0.05)
Deep IDA on top 50 selected features	0.63(0.10)	0.61(0.11)	0.70(0.10)	0.66(0.08)	0.75(0.16)	0.91(0.07)	0.90(0.08)	0.92(0.06)	0.91(0.08)	0.93(0.06)
Deep IDA view 1	**0.86**(0.09)	**0.85**(0.09)	**0.88**(0.07)	**0.86**(0.08)	**0.90**(0.07)	**0.94**(0.06)	**0.94**(0.06)	**0.94**(0.05)	**0.96**(0.05)	0.93(0.08)
Deep IDA	0.60(0.07)	0.59(0.07)	0.65(0.10)	0.66(0.08)	0.67(0.17)	0.85(0.07)	0.85(0.07)	0.87(0.06)	0.88(0.07)	0.86(0.07)
Deep CCA on top 50 selected features view 1	0.61(0.05)	0.61(0.05)	0.62(0.05)	0.69(0.05)	0.57(0.05)	0.57(0.03)	0.58(0.03)	0.59(0.03)	0.65(0.03)	0.55(0.03)
Deep CCA on top 50 selected features	0.58(0.04)	0.59(0.04)	0.60(0.05)	0.66(0.04)	0.56(0.06)	0.57(0.03)	0.58(0.03)	0.60(0.03)	0.65(0.03)	0.55(0.02)
Deep CCA view 1	0.59(0.04)	0.60(0.04)	0.61(0.04)	0.67(0.04)	0.56(0.05)	0.61(0.02)	0.61(0.02)	0.63(0.02)	0.68(0.02)	0.59(0.02)
Deep CCA	0.57(0.03)	0.58(0.03)	0.60(0.03)	0.65(0.04)	0.57(0.04)	0.60(0.02)	0.60(0.02)	0.62(0.02)	0.67(0.02)	0.58(0.02)
Sparse CCA with logistic regression view 1	0.58(0.01)	0.60(0.01)	0.43(0.01)	0.51(0.01)	0.37(0.01)	0.58(0.02)	0.60(0.01)	0.43(0.01)	0.51(0.01)	0.37(0.01)
Sparse CCA with logistic regression	0.58(0.01)	0.60(0.01)	0.43(0.01)	0.52(0.02)	0.37(0.01)	0.58(0.01)	0.60(0.01)	0.43(0.01)	0.51(0.01)	0.37(0.01)
PMA with logistic regression view 1	0.58(0.01)	0.60(0.01)	0.43(0.01)	0.51(0.01)	0.37(0.01)	0.58(0.01)	0.60(0.01)	0.43(0.01)	0.51(0.01)	0.37(0.01)
PMA with logistic regression	0.58(0.02)	0.60(0.01)	0.42(0.02)	0.52(0.04)	0.36(0.03)	0.58(0.01)	0.60(0.01)	0.43(0.01)	0.51(0.01)	0.37(0.01)
SVM on top 50 selected features view 1	0.50(0.01)	0.51(0.01)	0.52(0.01)	0.58(0.01)	0.47(0.01)	0.53(0.01)	0.53(0.01)	0.56(0.01)	0.60(0.01)	0.52(0.02)
SVM on top 50 selected features	0.50(0.01)	0.51(0.01)	0.52(0.01)	0.58(0.01)	0.47(0.02)	0.54(0.01)	0.54(0.01)	0.58(0.01)	0.61(0.01)	0.55(0.02)
SVM view 1	0.50(0.01)	0.51(0.01)	0.52(0.01)	0.58(0.01)	0.48(0.02)	0.53(0.01)	0.53(0.01)	0.56(0.01)	0.60(0.01)	0.52(0.02)
SVM	0.54(0.01)	0.54(0.01)	0.58(0.02)	0.61(0.02)	0.55(0.03)	0.54(0.01)	0.54(0.01)	0.58(0.01)	0.61(0.01)	0.55(0.02)

	Setting3	(*n*1 = 200,	*n*2 = 150,	*p*1 = 2000,	*p*2 = 2000)	Setting4	(*n*1 = 3000,	*n*2 = 2250,	*p*1 = 2000,	*p*2 = 2000)
Method	Accuracy	AUROC	*F*1	Precision	Recall	Accuracy	AUROC	*F*1	Precision	Recall
Deep IDA on top 200 selected features view 1	**0.71(0.07)**	**0.70(0.07)**	**0.74(0.07)**	0.74(0.06)	0.75(0.11)	0.70(0.06)	0.69(0.06)	0.74(0.06)	0.73(0.07)	0.77(0.12)
Deep IDA on top 200 selected features	0.58(0.04)	0.56(0.04)	0.67(0.04)	0.62(0.04)	0.74(0.11)	0.69(0.05)	0.67(0.05)	0.73(0.04)	0.72(0.05)	0.76(0.08)
Deep IDA view 1	0.53(0.07)	0.53(0.06)	0.56(0.15)	0.59(0.05)	0.58(0.22)	**0.73**(0.10)	**0.72**(0.11)	**0.78**(0.09)	**0.76**(0.11)	**0.81**(0.12)
Deep IDA	0.55(0.05)	0.51(0.02)	0.61(0.24)	0.53(0.18)	**0.80(0.34)**	0.67(0.07)	0.65(0.07)	0.72(0.06)	0.69(0.06)	0.75(0.07)
Deep CCA on top 200 selected features view 1	0.68(0.05)	0.69(0.05)	0.69(0.05)	0.77(0.05)	0.62(0.05)	0.62(0.08)	0.62(0.07)	0.64(0.08)	0.69(0.07)	0.60(0.10)
Deep CCA on top 200 selected features	0.61(0.03)	0.61(0.04)	0.63(0.03)	0.69(0.04)	0.58(0.03)	0.62(0.08)	0.62(0.07)	0.64(0.08)	0.69(0.07)	0.60(0.09)
Deep CCA view 1	0.70(0.02)	**0.70(0.02)**	0.71(0.02)	**0.78(0.03)**	0.66(0.03)	0.59(0.05)	0.60(0.05)	0.62(0.04)	0.67(0.05)	0.57(0.04)
Deep CCA	0.62(0.02)	0.63(0.02)	0.64(0.02)	0.70(0.03)	0.60(0.03)	0.61(0.03)	0.61(0.03)	0.64(0.02)	0.68(0.03)	0.60(0.03)
Sparse CCA with logistic regression view 1	0.58(0.01)	0.60(0.01)	0.43(0.01)	0.51(0.01)	0.37(0.01)	0.58(0.01)	0.60(0.01)	0.43(0.01)	0.51(0.01)	0.37(0.01)
Sparse CCA with logistic regression	0.57(0.01)	0.60(0.01)	0.43(0.01)	0.50(0.02)	0.37(0.01)	0.58(0.01)	0.60(0.01)	0.43(0.01)	0.51(0.01)	0.37(0.01)
PMA with logistic regression view 1	0.57(0.02)	0.60(0.01)	0.43(0.01)	0.50(0.03)	0.38(0.02)	0.58(0.01)	0.60(0.01)	0.43(0.01)	0.51(0.01)	0.37(0.01)
PMA with logistic regression	0.58(0.01)	0.60(0.01)	0.43(0.01)	0.51(0.01)	0.37(0.01)	0.58(0.01)	0.60(0.01)	0.43(0.01)	0.51(0.01)	0.37(0.01)
SVM on top 200 selected features view 1	0.50(0.02)	0.51(0.02)	0.52(0.02)	0.58(0.02)	0.46(0.03)	0.54(0.01)	0.54(0.01)	0.57(0.01)	0.61(0.01)	0.54(0.01)
SVM on top 200 selected features	0.51(0.02)	0.52(0.01)	0.53(0.02)	0.59(0.02)	0.48(0.03)	0.53(0.01)	0.53(0.01)	0.58(0.01)	0.60(0.01)	0.56(0.01)
SVM view 1	0.54(0.02)	0.54(0.02)	0.58(0.02)	0.61(0.01)	0.55(0.04)	0.54(0.01)	0.54(0.01)	0.57(0.01)	0.61(0.01)	0.54(0.01)
SVM	0.55(0.02)	0.53(0.02)	0.62(0.02)	0.59(0.02)	0.66(0.03)	0.53(0.01)	0.53(0.01)	0.58(0.01)	0.60(0.01)	0.56(0.01)

This table summarizes the evaluation of nonlinear simulations on the testing set across four distinct scenarios, showcasing metrics such as Accuracy, *F*1 Score, Precision, Recall, and AUROC curve. The top selected features are obtained by our proposed Deep IDA + Bi-Bootstrap. Each entry displays the mean values derived from 20 simulations, with the standard deviation provided in parentheses. All values rounded to 2 digits.

We bold highest values for each metric.

Taken together, the classification and variable selection accuracy from both the linear and nonlinear simulations suggest that the proposed method is capable of ranking the signal variables higher, and is also able to yield competitive or better classification performance, even in situations where the sample size is less than the number of variables.

### 3.2 Applications

#### 3.2.1 Overview

We consider two real datasets: (i) handwriting image data (analyzed in the Supplementary material) and (ii) COVID-19 multi-omics data. The image data was used to primarily assess the classification performance of the proposed method without feature ranking while the COVID-19 data was used to assess classification performance and to also demonstrate that Deep IDA is capable of identifying biologically relevant features.

For the analysis of data from the COVID-19 study, our goal is to elucidate the molecular architecture of COVID-19 severity by identifying molecular signatures that are associated with each other and can discriminate patients with and without COVID-19 who were or were not admitted to the ICU.

#### 3.2.2 Data preprocessing and application of deep IDA and competing methods

Of the 128 patients, 125 had both omics and clinical data. We focused on proteomics, RNA-seq, and metabolomics data in our analyses since many lipids were not annotated. We formed a four-class classification problem using COVID-19 and ICU status. Our four groups were: with COVID-19 and not admitted to the ICU (COVID Non-ICU), with COVID-19 and admitted to the ICU (COVID ICU), no COVID-19 and admitted to the ICU (Non-COVID ICU), and no COVID-19 and not admitted to the ICU (Non-COVID Non-ICU). The frequency distribution of samples in these four groups were: 40% COVID ICU, 40% COVID Non-ICU, 8% Non-COVID Non-ICU, and 12% Non-COVID ICU. The initial dataset contained 18, 212 genes, 517 proteins, and 111 metabolomics features. Prior to applying our method, we preprocessed the data (see Supplementary material) to obtain a final dataset of $X^{1}\in R^{125\times 2,734}$ for the RNA-sequencing data, $X^{2}\in R^{125\times 269}$ for the protoemics data, and $X^{3}\in R^{125\times 66}$ for the metabolomics. We randomly split the data into training (n=74) and testing (n=51) sets while keeping the proportions in each group similar to the original data, we applied the methods on the training data and we assessed error rate using the test data. We evaluated Deep IDA with and without feature selection. For Deep IDA with feature selection, we obtained the top 50 and top 10% molecules after implementing Algorithm 1, learned the model on the training data with only the molecules that were selected, and estimated the test error with the testing data. We also assessed the performance of the other methods using variables that were selected by Deep IDA. Supplementary Table S9 gives the network structure used.

Similar methods mentioned in Simulation are used for comparisons. The Sparse CCA is only for two views so cannot be applied here. We also compare Deep IDA to SIDA (Safo *et al.* 2021), a joint association and classification method that models linear relationships between views and among classes in each view.

#### 3.2.3 Deep IDA is better at discriminating patients with and without COVID-19 who were or were not admitted to the ICU

Supplementary Fig. S2 in the gives the top 50 genes, proteins, and metabolomics features that were highly ranked by Deep IDA. Feature importance for each variable was normalized to the feature ranked highest for each omics. Table 3 gives the balanced accuracy, weighted *F*1, weighted precision and weighted recall on test data for Deep IDA in comparison with Deep GCCA, SIDA, and SVM.

**Table 3.** Evaluating COVID-19 omics data on the testing set: balanced accuracy, weighted *F*1, precision, and recall.

Method	Balanced accuracy	Weighted *F*1	Weighted precision	Weighted recall
Deep IDA with top 100 selected features	0.76(0.08)	0.79(0.06)	0.81(0.06)	0.80(0.06)
Deep IDA with top 10% selected features	0.76(0.08)	0.79(0.07)	0.80(0.07)	0.79(0.07)
Deep IDA	0.70(0.07)	0.76(0.05)	0.77(0.06)	0.76(0.05)
Deep GCCA + SVM with top 10% selected features	0.55(0.16)	0.61(0.13)	0.66(0.12)	0.64(0.11)
Deep GCCA +SVM	0.61(0.14)	0.64(0.12)	0.68(0.10)	0.65(0.11)
Deep GCCA + NCC with top 10% selected features	0.60(0.12)	0.64(0.12)	0.68(0.11)	0.64(0.12)
Deep GCCA +NCC	0.67(0.08)	0.67(0.08)	0.70(0.08)	0.67(0.08)
SIDA	0.60(0.11)	0.67(0.10)	0.69(0.10)	0.67(0.11)
PMA + SVM	0.40(0.03)	0.56(0.04)	0.52(0.05)	0.62(0.05)
SVM with top 10% selected features	0.78(0.07)	0.82(0.05)	0.83(0.05)	0.82(0.05)
SVM	0.73(0.09)	0.78(0.06)	0.79(0.06)	0.78(0.06)
NCC with top 10% selected features	0.72(0.07)	0.72(0.06)	0.75(0.06)	0.72(0.06)
NCC	0.65(0.07)	0.67(0.05)	0.70(0.05)	0.67(0.05)

The top selected features are obtained by our proposed Deep IDA + Bi-Bootstrap. Each value is based on 20 random train-test splits of data. Mean value is followed by standard deviation in the parentheses.

We do not include methods only applicable to two views. The top 10% (i.e. 273 for view 1, 27 for view 2 and 7 for view 3) selected features refer to Deep IDA selected features based on the training set. SIDA and PMA + SVM incorporate their own feature selection mechanisms and thus utilize the entire datasets for training. Deep IDA, Deep GCCA, SVM, and NCC were trained using both the complete set of features and the top 10% of selected features. The results of Deep IDA from using the top 10% of, and top 100, variables, for feature selection were similar, albeit better than the test accuracy obtained with other methods, except for SVM applied on the selected features. We observed an improved test performance of 4−7% across all metrics for Deep IDA, SVM, and NCC when we trained these methods with selected features. The classification performance of Deep GCCA improved when a larger number of variables were included. Compared to SIDA, the joint association and classification method that assesses linear relationships in the views and among the groups, the proposed method has a higher test accuracy. Supplementary Fig. S3 gives the discriminant and correlation plots from Deep IDA based on the top-ranked 50 molecules from each omics. From the discriminant plots of the first three discriminant scores, we notice that the samples are well-separated in the training data. For the testing data, we observe some overlaps in the sample groups but the COVID ICU group seems to be separated from the COVID NON-ICU and NON-COVID ICU groups. This separation is more apparent in the RNA sequencing and proteomics data and less apparent in the metabolomics data. Further, based on the testing data, the correlation between the metabolomics and proteomics data was higher when considering the first and third discriminant scores (0.69 and 0.36), respectively. From the second discriminant score, the correlation between the RNA sequencing and proteomics data was higher (0.49). Overall, the mean correlation between the metabolomics and proteomics data was highest (0.4) while the mean correlation between the metabolomics and RNA sequencing data was lowest (0.09). These findings suggest that the proposed method is capable of modeling nonlinear relationships among the different views and groups, and has potential to identify features that can lead to better classification results.

#### 3.2.4 Strong enrichment for neurological, cancer, gastrointestinal, inflammatory, and metabolic diseases

We used the Ingenuity Pathway Analysis (IPA) software to investigate the molecular and cellular functions, pathways, and diseases enriched in the proteins, genes, and metabolites that were ranked in the top 50 by our variable selection method. For the top-ranked metabolomics features, we first used the MetaboAnalyst 5.0 (Pang *et al.* 2021) software to obtain their human metabolome database reference ID and then used IPA on the mapped data for function enrichment analysis. Of the top 50 ranked features, we were able to map 25 features.

We observed strong pathways, molecular and cellular functions, and disease enrichment (Supplementary Tables S4–S6). The top disease and disorders significantly enriched in our list of genes are found in Supplementary Table S5. We note that 36 of the biomolecules in our gene list were determined to be associated with neurological diseases. This finding aligns with studies that suggest that persons with COVID-19 are likely to have neurological manifestations such as reduced consciousness and stroke (Berlit *et al.* 2020, Taquet *et al.* 2021). Further, 48 genes from our list were determined to be associated with cancer. Again, this supports studies suggesting that individuals with immunocompromised system from cancer or individuals who recently recovered from cancer, for instance, are at higher risk for severe outcomes. As in our gene list, 34 proteins were determined to be associated with neurological disease. Other significantly enriched diseases in our protein list included infectious diseases (such as infection by SARS coronavirus), inflammatory response (such as inflammation of organ), and metabolic disease (including Alzheimer disease and diabetes mellitus). A recent review (Steenblock *et al.* 2021) found that up to 50% of those who have died from COVID-19 had metabolic and vascular disorders. In particular, patients with metabolic dysfunction (e.g. obesity, and diabetes) have an increased risk for developing severe COVID-19. Further, getting infected with SARS-CoV-2 can likely lead to new onset of diabetes. The top disease and disorders significantly enriched in our list of metabolites (Supplementary Table S4) included cancer and gastrointestinal disease (such as digestive system, and hepatocellular cancer). A recent article found that people with SARS-CoV-2 infection have increased risks of gastrointestinal disorders in the post-acute phase of COVID-19, and even evident in people who did not require hospitalization in the acute phase of COVID-19 infection Xu *et al.* (2023).

#### 3.2.5 Enriched pathways related to metabolic processes

**Figure 4.**
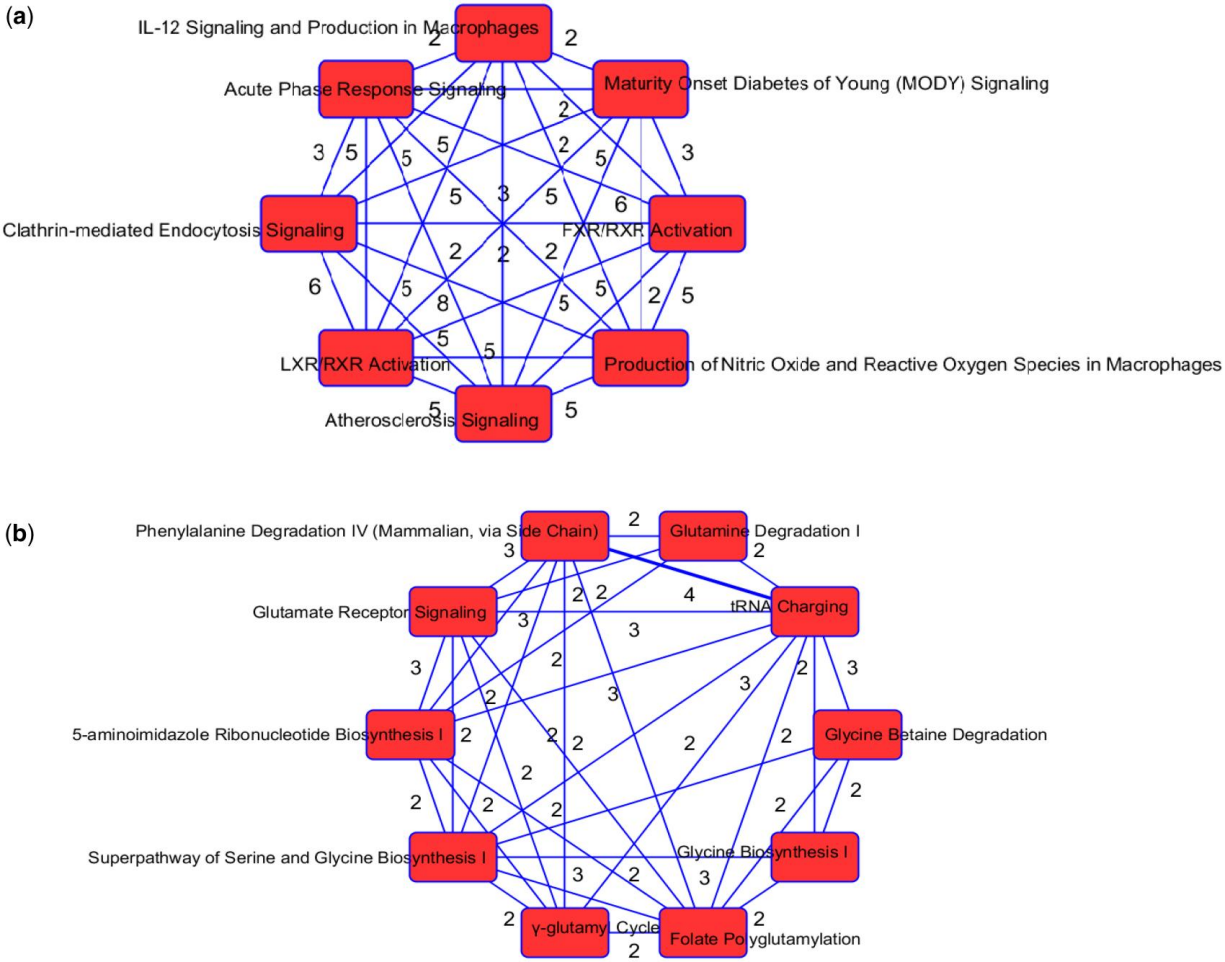
(a) Network of overlapping canonical pathways from highly ranked proteins. Nodes refer to pathways and a line connects any two pathways when there are at least two molecules in common between them. The pathways “FXR/RXR Activation” and “LXR/RXR Activation” share eight molecules: AGT, ALB, APOA2, APOH, APOM, CLU, PON1, and TF. (b) Network of overlapping canonical pathways from highly ranked metabolites. Nodes refer to pathways and a line connects any two pathways when there are at least two molecules in common between them.

The top enriched canonical pathways in our protein list include the LXR/RXR activation FXR/RXR activation, acute phase response signaling, and atherosclerosis signaling (Supplementary Table S4). These pathways are involved in metabolic processes such as cholesterol metabolism. The molecular and cellular functions enriched in our protein list include cellular movement and lipid metabolism (Supplementary Table S6). Overlapping canonical pathways (Fig. 4a and b) in IPA was used to visualize the shared biology in pathways through the common molecules participating in the pathways. The two pathways “FXR/RXR Activation” and “LXR/RXR Activation” share a large number (eight) of molecules (Fig. 4a) in our protein list: AGT, ALB, APOA2, APOH, APOM, CLU, PON1, and TF. The LXR/RXR pathway is involved in the regulation of lipid metabolism, inflammation, and cholesterol to bile acid catabolism. The farnesoid X receptor (FXR) is a member of the nuclear family of receptors and plays a key role in metabolic pathways and regulating lipid metabolism, cell growth and malignancy (Wang *et al.* 2008). We observed lower levels of ALB, APOM, and TF in patients with COVID-19 (and more so in patients with COVID-19 who were admitted to the ICU) relative to patients without COVID-19 (Supplementary Fig. S4). We also observed higher levels of AGT and CLU in patients with COVID-19 admitted to the ICU compared to the other groups. The fact that the top enriched pathways, and molecular and cellular functions are involved in metabolic processes such as lipid metabolism seem to corroborate the findings in Overmyer *et al.* (2021) that a key signature for COVID-19 is likely a dysregulated lipid transport system.

Taken together, these findings suggest that COVID-19 disrupts many biological systems. The relationships found with diseases such as cancer, gastrointestinal, neurological conditions, and metabolic diseases (e.g. Alzheimers and diabetes mellitus) heighten the need to study the post sequelae effects of this disease in order to better understand the mechanisms and to develop effective treatments and improve patient care.

## 4 Summary and conclusion

We have proposed a deep learning method, Deep IDA, for joint integrative analysis and classification studies of multiview data. Our framework extends the joint association and classification method proposed in Safo *et al.* (2021) to model complex nonlinear relationships among multiple views and between classes in a view.

The proposed algorithm, developed in Python 3, is user-friendly and will be useful in many data integration applications. Through simulation studies, we showed that the proposed method outperforms several other linear and nonlinear methods for integrating data from multiple views, even in high-dimensional scenarios where the sample size is typically smaller than the number of variables.

When Deep IDA was applied to proteomics, RNA sequencing, and metabolomics data obtained from individuals with and without COVID-19 who were or were not admitted to the ICU, we identified several molecules that better discriminated the COVID-19 patient groups. We also performed enrichment analysis of the molecules that were highly ranked and we observed strong pathways, molecular and cellular functions, and disease enrichment. Our findings have identified signatures that are related to neurological conditions, cancer, and metabolic diseases, corroborating current research findings and heightening the need to study the post sequelae effects of this disease to devise effective treatments and to improve patient care. The other top-ranked molecules from our findings could be further investigated to delineate their impact on COVID-19 status and severity.

Our proposed method has several strengths. First, by considering both relationships between views and separations between classes, our data integration process is improved, leading to better outcomes compared to approaches that focus solely on either association or classification. Second, unlike other deep learning models for discriminant analysis which have convergence issues, our loss is bounded and guarantees convergence. In our analyses, Deep IDA took about 20 epochs to converge. Third, our framework for feature ranking is general and applicable to other nonlinear methods for multiview data integration. Fourth, our algorithm is highly flexible, allowing us to handle different levels of data complexity by modifying the number of layers and nodes in the networks.

Our work has some limitations. First, the bootstrap technique proposed is computationally tasking. In our algorithm, we use parallelization to mitigate against the computational burden, however, more is needed to make the approach less expensive. Second, the proposed method has focused on binary or categorical outcomes. Future work could consider other outcome types (e.g. continuous and survival). Third, the number (or proportion) of top-ranked features need to be specified in advance. Third, the proposed loss function does not consider highly unbalanced classes. We can incorporate weighting, such as weighted LDA in Deep IDA’s model construction (1), to balance each class’s contribution. Fourth, the method is not designed for partial data, where some part of the samples does not have a measurement in one or more views. Missing data imputation can be considered in the future.

In this work, we applied the proposed method to integrate three multi-omics data, leading to biologically meaningful findings. As with any data integration method, a key question remains: whether more molecular data lead to better prediction performance and clinically meaningful findings. Although a holistic approach typically favors using more data, it is crucial to also consider the literature, biological significance, and clinical relevance of each molecular dataset in the study of the disease under consideration. Our decision to integrate these specific datasets was driven by the current literature and the need to understand the pathobiology of COVID-19. In fact, in the proposed method, one can assess the individual prediction performance of each view using the low-rank representations for that view; this will allow to investigate which views contribute most to the joint classification performance. It may be helpful to perturb the analysis by excluding views with poor classification performance.

In conclusion, we have developed a deep learning method to jointly model nonlinear relationships between multiple-view data and a binary or categorical outcome, while also producing highly ranked features contributing most to the overall association of the views and separation of the classes within a view. Despite the stated limitations, the encouraging simulations and real data findings, even for scenarios with small to moderate sample sizes, motivate further applications.

## Supplementary Material

vbae060_Supplementary_Data

## Data Availability

The gene expression, metabolomics, proteomics, lipidomics, and clinical data used are publicly available and details on how to obtain data are described in Overmyer *et al.* (2021). We provide a Python package, *Deep IDA*, to facilitate the use of our method. Its source codes, along with a README file, are available at: https://github.com/JiuzhouW/DeepIDA.
