# Supplementary material for: Deep IDA: a deep learning approach for integrative discriminant analysis of multi-omics data with feature ranking—an application to COVID-19
Source: Bioinform Adv. 2024 Apr 24;4(1):vbae060. doi: 10.1093/bioadv/vbae060 (PMC11256945; doi:10.1093/bioadv/vbae060)
Supplement: vbae060_Supplementary_Data [file vbae060_supplementary_data.pdf]

Supplementary Material for "Deep IDA: A Deep Learning Approach for Integrative Discriminant Analysis of Multi-omics Data with Feature Ranking- An Application to COVID-19"

1 More on Methods

1.1 Unique Features of Proposed Method

Property/ Methods	Linear Joint Methods Methods	Deep IDA (Proposed)	Randomized KCCA*	Deep CCA*, Deep GCCA+
Nonlinear Relationships		✓	✓	✓
Classification	✓	✓		
Variable ranking/selection	✓	✓		
Covariates	✓	✓		✓
Joint methods	✓	✓		

Table 1: Unique features of Deep IDA compared to other methods. *Only applicable to two views. +Covariates could be added as additional view in Deep GCCA. Joint Methods refere to methods that combine integration and classification simultaneously.

1.2 Integrative Discriminant Analysis (IDA) for joint association and classification

Let $\mathbf{S}_b^d$ and $\mathbf{S}_w^d$ be the between-class and within-class covariances for the d-th view, respectively. That is, $\mathbf{S}_b^d = \frac{1}{n-1}\sum_{k=1}^{K} n_k(\boldsymbol{\mu}_k^d - \boldsymbol{\mu}^d)(\boldsymbol{\mu}_k^d - \boldsymbol{\mu}^d)^{\mathrm{T}}$; $\mathbf{S}_w^d = \frac{1}{n-1}\sum_{k=1}^{K}\sum_{i=1}^{n}(\mathbf{x}_{ik}^d - \boldsymbol{\mu}_k)(\mathbf{x}_{ik}^d - \boldsymbol{\mu}_k^d)^{\mathrm{T}}$, and $\boldsymbol{\mu}_k^d = \frac{1}{n_k}\sum_{i=1}^{n_k}\mathbf{x}_{ik}^d$ is the mean for class k in view d. Let $\mathbf{S}_{dj}, j < d$ be the cross-covariance between the d-th and j-th views. Let $\mathcal{M}^d = \mathbf{S}_w^{d^{-1/2}}\mathbf{S}_b^d\mathbf{S}_w^{d^{-1/2}}$ and $\mathcal{N}_{dj} = \mathbf{S}_w^{d^{-1/2}}\mathbf{S}_{dj}\mathbf{S}_w^{j^{-1/2}}$. The IDA method finds linear discriminant vectors $(\widehat{\boldsymbol{\Gamma}}^1, \ldots, \widehat{\boldsymbol{\Gamma}}^d)$ that maximally associate the multiple views and separate the classes within each view by solving the optimization problem:

$$\max_{\boldsymbol{\Gamma}^1,\cdots,\boldsymbol{\Gamma}^D} \{\rho \sum_{d=1}^{D} \mathrm{tr}(\boldsymbol{\Gamma}^{d\mathrm{T}}\mathcal{M}^d\boldsymbol{\Gamma}^d) + \frac{2(1-\rho)}{D(D-1)} \sum_{d=1, d\neq j}^{D} \mathrm{tr}(\boldsymbol{\Gamma}^{d\mathrm{T}}\mathcal{N}_{dj}\boldsymbol{\Gamma}^j\boldsymbol{\Gamma}^{j\mathrm{T}}\mathcal{N}_{jd}\boldsymbol{\Gamma}^d)\} \text{ subject to } \mathrm{tr}(\boldsymbol{\Gamma}^{d\mathrm{T}}\boldsymbol{\Gamma}^d) = K-1. \quad (1)$$

The first term in equation (1) maximizes the sum of the separation of classes in each view, and the second term maximizes the sum of the squared pairwise correlations between two views. Here, ρ was used to control the influence of separation and association in the optimization problem. The optimum solution for equation (1) was shown to solve D systems of eigenvalue problem [12]. The discriminant loadings $(\widehat{\mathbf{\Gamma}}^1, \ldots, \widehat{\mathbf{\Gamma}}^d)$ correspond to the eigenvectors that maximally associate the views and separate the classes within each view. The views were projected onto the discriminant loadings to yield discriminant scores and samples were classified using nearest centroid. In the Method Section, we propose a novel nonlinear formulation of IDA using deep nueral networks (DNN) to study nonlinear associations among two or more views and separation of classes within a view. We also propose a homogeneous ensemble approach for feature ranking to identify features contributing most to the association of the views and the separation of the classes in a view.

1.3 Theorems and Proofs

Theorem 1. *Let $\mathbf{S}_t^d$ and $\mathbf{S}_b^d$ respectively be the total covariance and the between-class covariance for the top-level representations $\mathbf{H}^d, d = 1, \ldots, D$. Let $\mathbf{S}_{dj}$ be the cross-covariance between top-level representations d and j. Assume $\mathbf{S}_t^d \succ 0$. Define $\mathcal{M}^d = \mathbf{S}_t^{d^{-\frac{1}{2}}} \mathbf{S}_b^d \mathbf{S}_t^{d^{-\frac{1}{2}}}$ and $\mathcal{N}_{dj} = \mathbf{S}_t^{d^{-\frac{1}{2}}} \mathbf{S}_{dj} \mathbf{S}_t^{j^{-\frac{1}{2}}}$. Then $\mathbf{\Gamma}^d \in \Re^{o_d \times l}$, $l \leq \min\{K-1, o_1, \ldots, o_D\}$ in equation (4) of main text are eigenvectors corresponding respectively to eigenvalues $\mathbf{\Lambda}_d = diag(\lambda_{d_k}, \ldots, \lambda_{d_l})$, $\lambda_{d_k} > \cdots > \lambda_{d_l} > 0$ that iteratively solve the eigensystem problems:*

$$\left(c_1 \mathcal{M}^d + c_2 \sum_{j \neq d}^{D} \mathcal{N}_{dj} \mathbf{\Gamma}_j \mathbf{\Gamma}_j^T \mathcal{N}_{dj}^T \right) \mathbf{\Gamma}_d = \mathbf{\Lambda}_d \mathbf{\Gamma}_d, \forall d = 1, ..., D$$

where $c_1 = \frac{\rho}{D}$ and $c_2 = \frac{2(1-\rho)}{D(D-1)}$.

Prove. Solving the optimization problem is equivalent to iteratively solving the following generalized eigenvalue systems:

$$\left(c_1 \mathcal{M}^1 + c_2 \sum_{j=2}^{D} \mathcal{N}_{1j} \mathbf{\Gamma}_j \mathbf{\Gamma}_j^T \mathcal{N}_{1j}^T \right) \mathbf{\Gamma}_1 = \mathbf{\Lambda}_1 \mathbf{\Gamma}_1$$
$$\vdots$$
$$\left(c_1 \mathcal{M}^d + c_2 \sum_{j=1, j \neq d}^{D} \mathcal{N}_{dj} \mathbf{\Gamma}_j \mathbf{\Gamma}_j^T \mathcal{N}_{dj}^T \right) \mathbf{\Gamma}_d = \mathbf{\Lambda}_d \mathbf{\Gamma}_d$$
$$\vdots$$
$$\left(c_1 \mathcal{M}^D + c_2 \sum_{j=1}^{D-1} \mathcal{N}_{Dj} \mathbf{\Gamma}_j \mathbf{\Gamma}_j^T \mathcal{N}_{Dj}^T \right) \mathbf{\Gamma}_D = \mathbf{\Lambda}_D \mathbf{\Gamma}_D$$

where $c1 = \frac{\rho}{D}$ and $c2 = \frac{2(1-\rho)}{D(D-1)}$.

Proof. The Lagrangian is

$$\begin{aligned} L(\mathbf{\Gamma}_1, ..., \mathbf{\Gamma}_D, \lambda_1, ..., \lambda_D) &= \rho \frac{1}{D} \sum_{d=1}^{D} tr[\mathbf{\Gamma}_d^T \mathcal{M}^d \mathbf{\Gamma}_d] + (1-\rho) \frac{2}{D(D-1)} \sum_{d=1}^{D} \sum_{j, j \neq d}^{D} tr[\mathbf{\Gamma}_d^T \mathcal{N}_{dj} \mathbf{\Gamma}_j \mathbf{\Gamma}_j^T \mathcal{N}_{dj}^T \mathbf{\Gamma}_d] - \sum_{d=1}^{D} \eta_d (tr[\mathbf{\Gamma}_d^T \mathbf{\Gamma}_d] - l) \\ &= c_1 \sum_{d=1}^{D} tr[\mathbf{\Gamma}_d^T \mathcal{M}^d \mathbf{\Gamma}_d] + c_2 \sum_{d=1}^{D} \sum_{j, j \neq d}^{D} tr[\mathbf{\Gamma}_d^T \mathcal{N}_{dj} \mathbf{\Gamma}_j \mathbf{\Gamma}_j^T \mathcal{N}_{dj}^T \mathbf{\Gamma}_d] - \sum_{d=1}^{D} \lambda_d (tr[\mathbf{\Gamma}_d^T \mathbf{\Gamma}_d] - l) \qquad (2) \end{aligned}$$

The first order stationary solution for $\mathbf{\Gamma}_d (\forall d = 1, ..., D)$ is

$$\frac{\partial L(\mathbf{\Gamma}_1, ..., \mathbf{\Gamma}_D, \lambda_1, ..., \lambda_D)}{\partial \mathbf{\Gamma}_d^T} = 2c_1 \mathcal{M}^d \mathbf{\Gamma}_d + 2c_2 \sum_{j,j \neq d}^{D} (\mathcal{N}_{dj} \mathbf{\Gamma}_j \mathbf{\Gamma}_j^T \mathcal{N}_{dj}^T) \mathbf{\Gamma}_d - 2\lambda_d \mathbf{\Gamma}_d = \mathbf{0} \tag{3}$$

Rearranging the equation 3 we have

$$\left(c_1 \mathcal{M}^d + c_2 \sum_{j,j \neq d}^{D} \mathcal{N}_{dj} \mathbf{\Gamma}_j \mathbf{\Gamma}_j^T \mathcal{N}_{dj}^T \right) \mathbf{\Gamma}_d = \lambda_d \mathbf{\Gamma}_d$$

For $\mathbf{\Gamma}_j, \forall j \neq d$ fixed, the above can be solved for the eigenvalues of $(c_1 \mathcal{M}^d + c_2 \sum_{j,j \neq d}^{D} \mathcal{N}_{dj} \mathbf{\Gamma}_j \mathbf{\Gamma}_j^T \mathcal{N}_{dj}^T)$. Arrange the eigenvalues from large to small and denote $\mathbf{\Lambda}_d \in \mathbf{R}^{o_d \times o_d}$ as the diagonal matrix of those values. For the top l largest eigenvalues, denote the corresponding eigenvectors as $\widehat{\mathbf{\Gamma}}_d = [\gamma_{d,1}, ..., \gamma_{d,l}]$. Therefore, starting from $d = 1$, following this process, $\widehat{\mathbf{\Gamma}}_1$ is updated; then, update $\widehat{\mathbf{\Gamma}}_2$ and so on; finally, update $\widehat{\mathbf{\Gamma}}_D$. We iterate until convergence, which is defined as, $\frac{\|\widehat{\mathbf{\Gamma}}_{d,new} - \widehat{\mathbf{\Gamma}}_{d,old}\|_F^2}{\|\widehat{\mathbf{\Gamma}}_{d,old}\|_F^2} < \epsilon$. When convergence is achieved, set $\widetilde{\mathbf{\Gamma}}_d = \widehat{\mathbf{\Gamma}}_d, \forall d = 1, ..., D$. ■

Theorem 2. *For d fixed, let $\eta_{d,1}, \ldots, \eta_{d,l}$, $l \leq \min\{K-1, o_1, \ldots, o_D\}$ be the largest l eigenvalues of $c_1 \mathcal{M}^d + c_2 \sum_{j \neq d}^{D} \mathcal{N}_{dj} \mathbf{\Gamma}_j \mathbf{\Gamma}_j^T \mathcal{N}_{dj}^T$. Then the solution $\widetilde{f}^d$ to the optimization problem in equation (5) [main text] for view d maximizes*

$$\sum_{r=1}^{l} \eta_{d,r}. \tag{4}$$

Proof. Fix d and let $\eta_{d,1}, \eta_{d,2}, ..., \eta_{d,l}$ be the top l eigenvalues of

$$c_1 \mathcal{M}^d + c_2 \sum_{j \neq d}^{D} \mathcal{N}_{dj} \widetilde{\mathbf{\Gamma}}_j \widetilde{\mathbf{\Gamma}}_j^T \mathcal{N}_{dj}^T.$$

Then,

$$\sum_{r=1}^{l} \eta_{d,r} = c_1 tr[\widetilde{\mathbf{\Gamma}}_d^T \mathcal{M}^d \widetilde{\mathbf{\Gamma}}_d] + c_2 \sum_{j,j \neq d}^{D} tr[\widetilde{\mathbf{\Gamma}}_d^T \mathcal{N}_{dj} \widetilde{\mathbf{\Gamma}}_j \widetilde{\mathbf{\Gamma}}_j^T \mathcal{N}_{dj}^T \widetilde{\mathbf{\Gamma}}_d]$$

Proof.

$$\begin{aligned}
& c_1 tr[\widetilde{\mathbf{\Gamma}}_d^T \mathcal{M}^d \widetilde{\mathbf{\Gamma}}_d] + c_2 \sum_{j,j \neq d}^{D} tr[\widetilde{\mathbf{\Gamma}}_d^T \mathcal{N}_{dj} \widetilde{\mathbf{\Gamma}}_j \widetilde{\mathbf{\Gamma}}_j^T \mathcal{N}_{dj}^T \widetilde{\mathbf{\Gamma}}_d] \\
&= tr(\widetilde{\mathbf{\Gamma}}_d^T (c_1 \mathcal{M}^d + c_2 \sum_{j,j \neq d}^{D} \mathcal{N}_{dj} \widetilde{\mathbf{\Gamma}}_j \widetilde{\mathbf{\Gamma}}_j^T \mathcal{N}_{dj}^T) \widetilde{\mathbf{\Gamma}}_d) \\
&= tr(\widetilde{\mathbf{\Gamma}}_d^T \mathbf{\Lambda}_d \widetilde{\mathbf{\Gamma}}_d) \\
&= \sum_{r=1}^{l} \eta_{d,r}
\end{aligned}$$

■

1.4 Comparison of Deep IDA with some existing nonlinear methods

Unlike Deep CCA [1] and Deep generalized CCA [2] which use DNN networks to learn transformations of nonlinearly transformed views to maximize associations between two (Deep CCA) or more (Deep generalized CCA) views, we maximize both association of views and separation of classes in a view jointly, thus we do not

need any sophisticated classification algorithm which could add another layer of computational complexity. Our proposed method is closely related to the method (multiview linear discriminant analysis network, MvLDAN in short) in [5] since we too find linear projections of nonlinearly transformed views that separate classes within each view and maximize correlation between multiple views. In [5], the authors proposed to solve the following optimization problem for linear projection matrices $\mathbf{A}_1, \cdots, \mathbf{A}_D$ and neural network parameters (weights and biases):

$$\underset{f^1,\cdots,f^D,\mathbf{A}_1,\cdots,\mathbf{A}_D}{\operatorname{argmax}} \quad tr\left((\mathbf{S}_w + \beta \mathbf{A}^{\mathrm{T}}\mathbf{A})^{-1}(\mathbf{S}_b + \lambda \mathbf{S}_c)\right), \tag{5}$$

where $\mathbf{A} = [\mathbf{A}_1^{\mathrm{T}} \cdots \mathbf{A}^{D^{\mathrm{T}}}]^{\mathrm{T}}$ is a concatenation of projection matrices from all views, $\mathbf{S}_b$ and $\mathbf{S}_w$ are the between-class and within-class covariances for all views, respectively, and $\mathbf{S}_c$ is the cross-covariance matrix for all views. Further, λ and β are regularization parameters. The authors then considered to learn the parameters of the deep neural network by maximizing the smallest eigenvalues of the generalized eigenvalue problem arising from equation (5) that do not exceed some threshold that is specified in advance. Although we have the same goal, our optimization formulation in equation (2) of the main text, and our loss function is different. We constrain the total covariance matrix $\mathbf{S}_t$ instead of $\mathbf{S}_w$ and as noted above, our loss function is bounded. As such, we do not have convergence issues when training our deep learning parameters. Deep LDA ([3]), a deep neural network-based linear discriminant analysis, suffers from this convergence issue. A major drawback of MvLDAN, Deep CCA, Deep generalized CCA and most existing DNN methods is that they cannot be used to identify variables contributing most to the association among the views and/or separation in the classes. Recently, a DNN method for multiview multi-task classification problems, MOMA, that uses attention mechanism for interpretability has been proposed [10]. We note that although MOMA is developed for two or more views, the algorithm the authors provide is applicable to only two views.

1.5 Optimization and Algorithm

The training process of DeepIDA is as follows:

- Feed forward and calculate the loss. The output for D deep neural networks are $\mathbf{H}^1, ..., \mathbf{H}^D$, which includes the neural network parameters (i.e., the weights and biases). Based on the objective in equation (6) of the main text, the final loss is calculated and denoted as $\mathcal{L} = -\sum_{d=1}^{D}\sum_{r=1}^{l}\eta_{d,r}$.

- Gradient of the loss function. The loss function $\mathcal{L}$ depends on the estimated linear projections $\widetilde{\mathbf{\Gamma}}^d, d = 1, \cdots, D$ and since these linear projections use the outputs of the last layer of the network, there are no parameters involved. Therefore, we calculate the gradient of the loss with respect to the view-specific output, i.e., $\frac{\partial \mathcal{L}}{\partial \mathbf{H}^d}, d = 1, \ldots, D$.

- Gradient within each sub-network. Since each view-specific sub-network is propagated separately, we can calculate the gradient of each sub-network independently. As the neural network parameters (i.e., weights and biases) of view d network is denoted as θ^d, we can calculate the partial derivative of last layer with respect to sub-network parameters as $\frac{\partial \mathbf{H}^d}{\partial \theta^d}$. These networks include shallow or multiple layers and nonlinear activation functions, such as Leaky-ReLu [8].

- Deep IDA update via gradient descent. By the chain rule, we can calculate $\nabla_{\theta^d}\mathcal{L} = \frac{\partial \mathcal{L}}{\partial \mathbf{H}^d} \cdot \frac{\partial \mathbf{H}^d}{\partial \theta^d}$. We use the *autograd* function in the PyTorch [11] package to compute this gradient. Therefore, for every

optimization step, a stochastic gradient descent-based optimizer, such as Adam [6], is used to update the network parameters.

We describe the Deep IDA algorithm in Algorithm 1. We also describe in Algorithm 2 the approach for obtaining the linear projections using the output of the final layer.

Algorithm 1 Algorithm of Deep IDA

Input: Training data $\mathbf{X} = \{\mathbf{X}^1, \mathbf{X}^2, ..., \mathbf{X}^D\}$ and class labels $\mathbf{y}$; number of nodes of each layer in D neural networks (including dimensions of linear sub-spaces to project onto, $o_1, o_2, ..., o_D$); learning rate α

Output: Optimized weights and biases for D neural networks and corresponding estimates $(\widetilde{f^1}(\mathbf{X}^1), ..., \widetilde{f^D}(\mathbf{X}^D))$

Initialization Assign random numbers to weights and biases for D neural networks

while loss not converge **do**

  Feed forward the network of each view with latest weights and biases to obtain the final layer $\mathbf{H}^d = f^d(\mathbf{X}^d) \in \mathbf{R}^{n \times o_d}, d = 1, 2, .., D$

  Apply Algorithm 2 to obtain $\widetilde{\mathbf{\Gamma}}_1, ..., \widetilde{\mathbf{\Gamma}}_D$

  Compute eigenvalues of $c_1 \mathcal{M}^d + c_2 \sum_{j, j \neq d}^{D} \mathcal{N}_{dj} \widetilde{\mathbf{\Gamma}}_j \widetilde{\mathbf{\Gamma}}_j^{\mathrm{T}} \mathcal{N}_{dj}^{\mathrm{T}}$ to obtain the loss function $-\sum_{d=1}^{D} \sum_{i=1}^{l} \eta_{d,i}$

  Compute the gradient of weights and biases for each network by the PyTorch *Autograd* function

  Update the weights and biases with the specified learning rate α

end while

Algorithm 2 Algorithm for iteratively solving Eigenvalue Problem

---

Input: Training data $\mathbf{H} = \{\mathbf{H}^1, \mathbf{H}^2, ..., \mathbf{H}^D\}$ and corresponding class labels $\mathbf{y}$; convergence criteria ϵ

Output: Optimized discriminant loadings $\widetilde{\mathbf{\Gamma}}_1, ..., \widetilde{\mathbf{\Gamma}}_D$

Compute $\mathcal{M}^d$, $\mathcal{N}_{dj}$ for $d, j = 1, 2, .., D$

while $\max_{d=1,..,D} \frac{\|\widehat{\mathbf{\Gamma}}_{d,new} - \widehat{\mathbf{\Gamma}}_{d,old}\|_F^2}{\|\widehat{\mathbf{\Gamma}}_{d,old}\|_F^2} > \epsilon$ **do**

  for $d = 1, .., D$ **do**: fix $\widehat{\mathbf{\Gamma}}_j, \forall j \neq d$, compute $\widehat{\mathbf{\Gamma}}_d$ by **Theorem 1**

end while

Set $\widetilde{\mathbf{\Gamma}}_d = \widehat{\mathbf{\Gamma}}_d, \forall d = 1, ..., D$

1.6 Run Time

For a desktop with memory = 32G, one single run of DeepIDA usually takes two minutes until convergence (around 50 epochs) on data with sample size 6000 and feature size 2000. In addition, the feature ranking process for one bootstrap averages around 4 hours for data of dimension 6000 × 2000. However, with parallel computing, it's possible to train 50 bootstraps concurrently within the same timeframe. For datasets with smaller sample sizes, like those in our real data analysis, a single Deep IDA run takes less than 10 seconds. The total time for feature ranking based on 50 bootstraps is approximately 1.5 hours when using 15-core parallel computing.

2 Simulations

2.1 Set-up

We conduct simulation studies to assess the performance of Deep IDA for varying data dimensions, and as the relationship between the views and within a view become more complex. We demonstrate that Deep IDA is capable of i) simultaneous association of data from multiple views and discrimination of sample classes, and ii) identifying signal variables.

We consider two different simulation Scenarios. In Scenario One, we simulate data to have linear relationships between views and linear decision boundaries between classes (Refer to Supplementary Material for set-up and results). In Scenario Two, we simulate data to have nonlinear relationships between views and nonlinear decision boundaries between classes. There are $K = 3$ classes and $D = 2$ and $D = 3$ views in Scenario One. In Scenario Two, there are $K = 2$ classes and $D = 2$ views. In all Scenarios, we generate 20 Monte Carlo training, validation, and testing sets. We evaluate the proposed and existing methods using the following criteria: i) test accuracy, and ii) feature selection. For feature selection, we evaluate the methods ability to select the true signals. In Scenario One, the first 20 variables are important, and in Scenario Two, the Top 10% of variables in view 1 are signals. Since Deep IDA and the teacher-student (TS) framework rank features, and SIDA assigns zero weights to unimportant variables, for fair comparison, we only assess the number of signal variables that are in the Top 20 (for Scenario One) and the Top 10% (for Scenario Two) variables selected by the methods. We compare test accuracy for Deep IDA with and without the variable ranking approach proposed in this manuscript.

2.2 Comparison Methods

We compare Deep IDA with classification-, association-, and joint association and classification-based methods. For classification-based methods, we consider the support vector machine [4] on stacked views. For association-based methods, we consider nonlinear methods such as deep canonical correlation analysis (Deep CCA) [1] and deep generalized CCA (DGCCA) [2] when there are three or more views. The association-based methods only consider nonlinear associations between views, as such we follow with SVM to perform classification using the learned low-dimensional representations from the methods. We also compare Deep IDA to SIDA [12], a joint association and classification method that models linear relationships between views and among classes in each view. We perform SIDA using the Matlab codes the authors provide. We perform Deep CCA and DGCCA using the PyTorch codes the authors provide. We couple Deep CCA and DGCCA with the teacher-student framework (TS) [9] to rank variables. We also investigate the performance of these methods when we use variables selected from Deep IDA.

2.3 Linear Simulations

We consider two simulation settings in this Scenario and we simulate data similar to simulations in [12]. In Setting One, there are $D = 2$ views $\mathbf{X}^1$ and $\mathbf{X}^2$, with $p_1 = 1,000$ and $p_2 = 1,000$ variables respectively. There are $K = 3$ classes each with size $n_k = 180$, $k = 1,2,3$ giving a total sample size of $n = 540$. Let $\mathbf{X}^d = [\mathbf{X}_1^d, \mathbf{X}_2^d, \mathbf{X}_3^d], d = 1,2$ be a concatenation of data from the three classes. The combined data $\left(\mathbf{X}_k^1, \mathbf{X}_k^2\right)$ for each class are simulated from $N(\boldsymbol{\mu}_k, \boldsymbol{\Sigma})$, $\boldsymbol{\mu}_k = (\boldsymbol{\mu}_k^1, \boldsymbol{\mu}_k^2)^{\mathrm{T}} \in \Re^{p_1+p_2}, k = 1,2,3$ is the combined mean vector for class k; $\boldsymbol{\mu}_k^1 \in \Re_1^p, \boldsymbol{\mu}_k^2 \in \Re_2^p$ are the mean vectors for $\mathbf{X}_k^1$ and $\mathbf{X}_k^2$ respectively. The covariance matrix $\boldsymbol{\Sigma}$ for the

combined data for each class is partitioned as

$$\mathbf{\Sigma} = \begin{pmatrix} \mathbf{\Sigma}^1 & \mathbf{\Sigma}^{12} \\ \mathbf{\Sigma}^{21} & \mathbf{\Sigma}^2 \end{pmatrix}, \mathbf{\Sigma}^1 = \begin{pmatrix} \widetilde{\mathbf{\Sigma}}^1 & \mathbf{0} \\ \mathbf{0} & \mathbf{I}_{p_1-20} \end{pmatrix}, \mathbf{\Sigma}^2 = \begin{pmatrix} \widetilde{\mathbf{\Sigma}}^2 & \mathbf{0} \\ \mathbf{0} & \mathbf{I}_{p_2-20} \end{pmatrix}$$

where $\mathbf{\Sigma}^1$, $\mathbf{\Sigma}^2$ are respectively the covariance of $\mathbf{X}^1$ and $\mathbf{X}^2$, and $\mathbf{\Sigma}^{12}$ is the cross-covariance between the two views. We generate $\widetilde{\mathbf{\Sigma}}^1$ and $\widetilde{\mathbf{\Sigma}}^2$ as block diagonal with 2 blocks of size 10, between-block correlation 0, and each block is a compound symmetric matrix with correlation 0.8. We generate the cross-covariance matrix $\mathbf{\Sigma}^{12}$ as follows. Let $\mathbf{V}^1 = [\mathbf{V}^1_1,\ \mathbf{0}_{(p_1-20)\times 2}]^{\mathrm{T}} \in \Re^{p_1\times 2}$ and the entries of $\mathbf{V}^1_1 \in \Re^{20\times 2}$ are *i.i.d* samples from U(0.5,1). We similarly define $\mathbf{V}^2$ for the second view, and we normalize such that $\mathbf{V}^{1^T}\mathbf{\Sigma}^1\mathbf{V}^1 = \mathbf{I}$ and $\mathbf{V}^{2^{\mathrm{T}}}\mathbf{\Sigma}^2\mathbf{V}^2 = \mathbf{I}$. We then set $\mathbf{\Sigma}^{12} = \mathbf{\Sigma}^1\mathbf{V}^1\mathbf{D}\mathbf{V}^{2^{\mathrm{T}}}\mathbf{\Sigma}^2$, $\mathbf{D} = \mathrm{diag}(0.4, 0.2)$ to depict moderate association between the views. For class separation, define the matrix $[\mathbf{\Sigma}\mathbf{A}, \mathbf{0}_{(p_1+p_2)}] \in \Re^{(p_1+p_2)\times 3}$; $\mathbf{A} = [\mathbf{A}^1, \mathbf{A}^2]^{\mathrm{T}} \in \Re^{(p_1+p_2)\times 2}$, and set the first, second, and third columns as the mean vector for class 1, 2, and 3, respectively. Here, the first column of $\mathbf{A}^1 \in \Re^{p_1\times 2}$ is set to $(c\mathbf{1}_{10}, \mathbf{0}_{p_1-10})$ and the second column is set to $(\mathbf{0}_{10}, -c\mathbf{1}_{10}, \mathbf{0}_{p-20})$; we fix c at 0.2. We set $\mathbf{A}^2 \in \Re^{p_2\times 2}$ similarly, but we fix c at 0.1 to allow for different class separation in each view.

In Setting Two, we simulate $D = 3$ views, $\mathbf{X}^d, d = 1, 2, 3$, and each view is a concatenation of data from three classes as before. The combined data $\left(\mathbf{X}^1_k, \mathbf{X}^2_k, \mathbf{X}^3_k\right)$ for each class are simulated from $N(\boldsymbol{\mu}_k, \mathbf{\Sigma})$, where $\boldsymbol{\mu}_k = (\boldsymbol{\mu}^1_k, \boldsymbol{\mu}^2_k, \boldsymbol{\mu}^3_k)^{\mathrm{T}} \in \Re^{p_1+p_2+p_3}, k = 1, 2, 3$ is the combined mean vector for class k; $\boldsymbol{\mu}^d_k \in \Re^{p_d}, j = 1, 2, 3$ are the mean vectors for $\mathbf{X}^d_k, d = 1, 2, 3$. The true covariance matrix $\mathbf{\Sigma}$ is defined similar to Setting One but with the following modifications. We include $\mathbf{\Sigma}_3$, $\mathbf{\Sigma}_{13}$, and $\mathbf{\Sigma}_{23}$, and we set $\mathbf{\Sigma}_{13} = \mathbf{\Sigma}_{23} = \mathbf{\Sigma}_{12}$. Like $\mathbf{\Sigma}_1$ and $\mathbf{\Sigma}_2$, $\mathbf{\Sigma}_3$ is partitioned into signals and noise, and the covariance for the signal variables, $\widetilde{\mathbf{\Sigma}}^3$, is also block diagonal with 2 blocks of size 10, between-block correlation 0, and each block is compound symmetric matrix with correlation 0.8. We take $\boldsymbol{\mu}_k$ to be the columns of $[\mathbf{\Sigma}\mathbf{A}, \mathbf{0}_{(p_1+p_2+p_3)}] \in \Re^{(p_1+p_2+p_3)\times 2}$, and $\mathbf{A} = [\mathbf{A}^1, \mathbf{A}^2, \mathbf{A}^3]^{\mathrm{T}} \in \Re^{(p_1+p_2+p_3)\times 2}$. The first column of $\mathbf{A}^j \in \Re^{p_j\times 2}$ is set to $(c_j\mathbf{1}_{10}, \mathbf{0}_{p_1-10})$ and the second column is set to $(\mathbf{0}_{10}, -c_j\mathbf{1}_{10}, \mathbf{0}_{p-20})$ for $j = 1, 2, 3$. We set $(c_1, c_2, c_3) = (0.2, 0.1, 0.05)$ to allow for different class separation in each view.

2.3.1 Results for Linear Simulations

Table 2 gives classification accuracy for the methods and the true positive rates for the top 20 variables selected. We implemented a three-hidden layer network with dimensions 512, 256, and 64 for both Deep IDA and Deep CCA. The dimension of the output layer was set as 10. Table 7 lists the network structure used for each setting. For Deep IDA + Bootstrap, the bootstrap algorithm proposed in the Methods Section was implemented on the training data to choose the top 20 ranked variables. We then implemented Deep IDA on the training data but with just the variables ranked in the top 20 in each view. The learned model and the testing data were used to obtain the test errors. To compare our feature ranking process with the teacher-student (TS) network approach for feature ranking, we also implemented Deep IDA without the bootstrap approach for feature ranking, and we used the learned model from Deep IDA in the TS framework for feature ranking. We also performed feature ranking using the learned model from Deep CCA (Setting One) and Deep GCCA (Setting Two). The average error rates for the nonlinear methods are higher than the error rate for SIDA, a linear method for joint association and classification analysis. This is not surprising as the true relationships among the views, and the classes within a view are linear. Nevertheless, the average test error rate for Deep IDA that is based on the top 20 variables in each view from the bootstrap method (i.e., Deep IDA + Bootstrap) is lower than the average test error rates from Deep CCA and SVM (on stacked views). When we implemented Deep CCA, SVM,

and DGCCA on the top 20 variables that were selected by our proposed method, we observed a decrease in the error rate across the methods. For instance, the error rates for Deep CCA using all variables compared to using the top 20 variables identified by our method were 33.17% and 22.95%, respectively. Further, compared to Deep IDA on all variables (i.e., Deep IDA + TS), Deep IDA + Bootstrap has a lower average test error, demonstrating the advantage of variable selection. In Setting Two, the classification accuracy for Deep GCCA was poor. In terms of variable selection, compared to SIDA, the proposed method was competitive at identifying the top-ranked 20 variables. The TS framework for ranking variables was sub-optimal as evident from the true positive rates for Deep IDA + TS, Deep CCA + TS, and Deep GCCA + TS.

Table 2: Linear Setting: RS; randomly select tuning parameters space to search. TPR-1; true positive rate for $\mathbf{X}^1$. Similar for TPR-2. TS: Teacher student network. $-$ indicates not applicable. Deep IDA on selected top 20 variables is when we use the bootstrap algorithm to choose the top 20 ranked variables, train Deep IDA with the top 20-ranked variables, and then use the learned model and the test data to obtain test errors.

Method	Error (%)	TPR-1	TPR-2	TPR-3
**Setting One**				
Deep IDA on selected top 20 variables	24.69	100.00	95.25	-
Deep IDA+ TS	33.87	33.25	21.75	-
SIDA	20.81	99.50	93.50	-
Deep CCA + TS	33.17	4.25	3.25	-
Deep CCA on selected top 20 variables	22.95	-	-	-
SVM (Stacked views)	31.53	-	-	-
SVM on selected top 20 variables (Stacked views)	22.03	-	-	-
**Setting Two**				
Deep IDA on selected top 20 variables	23.16	100.00	94.75	78.75
Deep IDA + TS	31.22	72.00	57.75	47.75
SIDA	19.79	99.75	99.50	97.25
DGCCA + TS	60.01	2.0	2.0	2.25
DGCCA on selected top 20 variables	57.40	-	-	-
SVM (Stacked views)	29.06	-	-	-
SVM on selected top 20 variables (Stacked views)	19.56	-	-	-

3 More Results From Real Data Analysis

3.1 Evaluation of the Noisy MNIST digits data

The original MNIST handwritten image dataset [7] consists of 70,000 grayscale images of handwritten digits split into training, validation and testing sets of 50,000, 10,000 and 10,000 images, respectively. The validation set was used to select network parameters from the best epoch (lowest validation loss). Each image is 28×28 pixels and has associated with it a label that denotes which digit the image represents (0-9). In [13], a more complex and challenging noisy version of the original data was generated and used as a second view. First, all pixel values were scaled to 0 and 1. The images were randomly rotated at angles uniformly sampled from $[-\frac{\pi}{4}, \frac{\pi}{4}]$, and the resulting images were used as view 1. Each rotated image was paired with an image of the same label from the original MNIST data, independent random noise generated from U(0,1) was added, and the pixel values were truncated to [0,1]. The transformed data is view 2. Figure 1 shows two image plots of a digit for views 1 and 2. Of note, view 1 is informative for classification and view 2 is noisy. Therefore, an ideal multiview classification method should be able to extract the useful information from view 1 while disregarding the noise in view 2.

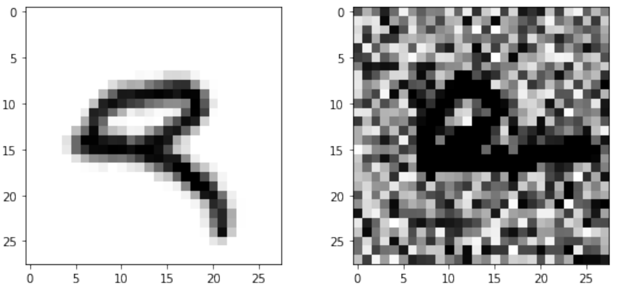

Figure 1: An example of Noisy MNIST data. For the subject with label ”9”, view 1 observation is on the left and view 2 observation is on the right.

The goal of this application is to evaluate how well the proposed method without feature ranking can classify the digits using the two views. Thus, we applied Deep IDA without feature ranking and the competing methods to the training data and we used the learned models and the testing data to obtain test classification accuracy. The validation data was used in Deep IDA and Deep CCA to choose the best model among all epochs. Table 9 in the supplementary material lists the network structure used in this analysis. Table 3 gives the test classification results of the methods. We observe that the test classification accuracy of the proposed method with nearest centroid classification is better than SVM on the stacked data, and is comparable to Deep CCA. We observe a slight advantage of the proposed method when we implement SVM on the final layer of Deep IDA.

Table 3: Noisy MNIST data: SVM was implemented on the stacked data. For Deep CCA + SVM, we trained SVM on the combined outputs (from view 1 and view 2) of the last layer of Deep CCA. For Deep IDA + NCC, we implemented the Nearest Centroid Classification on the combined outputs (from view 1 and view 2) of the last layer of Deep IDA. For Deep IDA + SVM, we trained SVM on the combined outputs (from view 1 and view 2) of the last layer of Deep IDA.

Method	Accuracy (%)
SVM (combined view 1 and 2)	93.75
Deep CCA + SVM	97.01
Deep IDA + NCC	97.74
Deep IDA + SVM	99.15
RKCCA + SVM	91.79

3.2 Data pre-processing and application of Deep IDA and competing methods

Of the 128 patients, 125 had both omics and clinical data. We focused on proteomics, RNA-seq, and metabolomics data in our analyses since many lipids were not annotated. We formed a four-class classification problem using COVID-19 and ICU status. Our four groups were: with COVID-19 and not admitted to the ICU (COVID Non-ICU), with COVID-19 and admitted to the ICU (COVID ICU), no COVID-19 and admited to the ICU (Non-COVID ICU), and no COVID-19 and not admitted to the ICU (Non-COVID Non-ICU). The frequency distribution of samples in these four groups were: 40% COVID ICU, 40% COVID Non-ICU, 8% Non-COVID Non-ICU, and 12% Non-COVID ICU. The initial dataset contains 18,212 genes, 517 proteins, and 111 metabolomics features. Prior to applying our method, we pre-processed the data as follows. All genes which were missing in our samples were removed from the dataset and 15,106 genes remained. We selected genes that more than half

of their variables are non-zero, and we applied box-cox transformation on each gene as the gene data were highly skewed. The transformed data were standardized to have mean zero and variance one. We kept genes with variance less than the 25th percentile. We then used ANOVA on the standardized data to filter out (p-values > 0.05) genes with low potential to discriminate among the four groups. For the proteomics and metabolomics data, we standardized each molecule to have mean zero and variance one, pre-screened with ANOVA and filtered out molecules with p-values > 0.05. Our final data were $\mathbf{X}^1 \in \Re^{125\times 2,734}$ for the gene data, $\mathbf{X}^2 \in \Re^{125\times 269}$ for the protoemics data, and $\mathbf{X}^3 \in \Re^{125\times 66}$ for the metabolomics data.

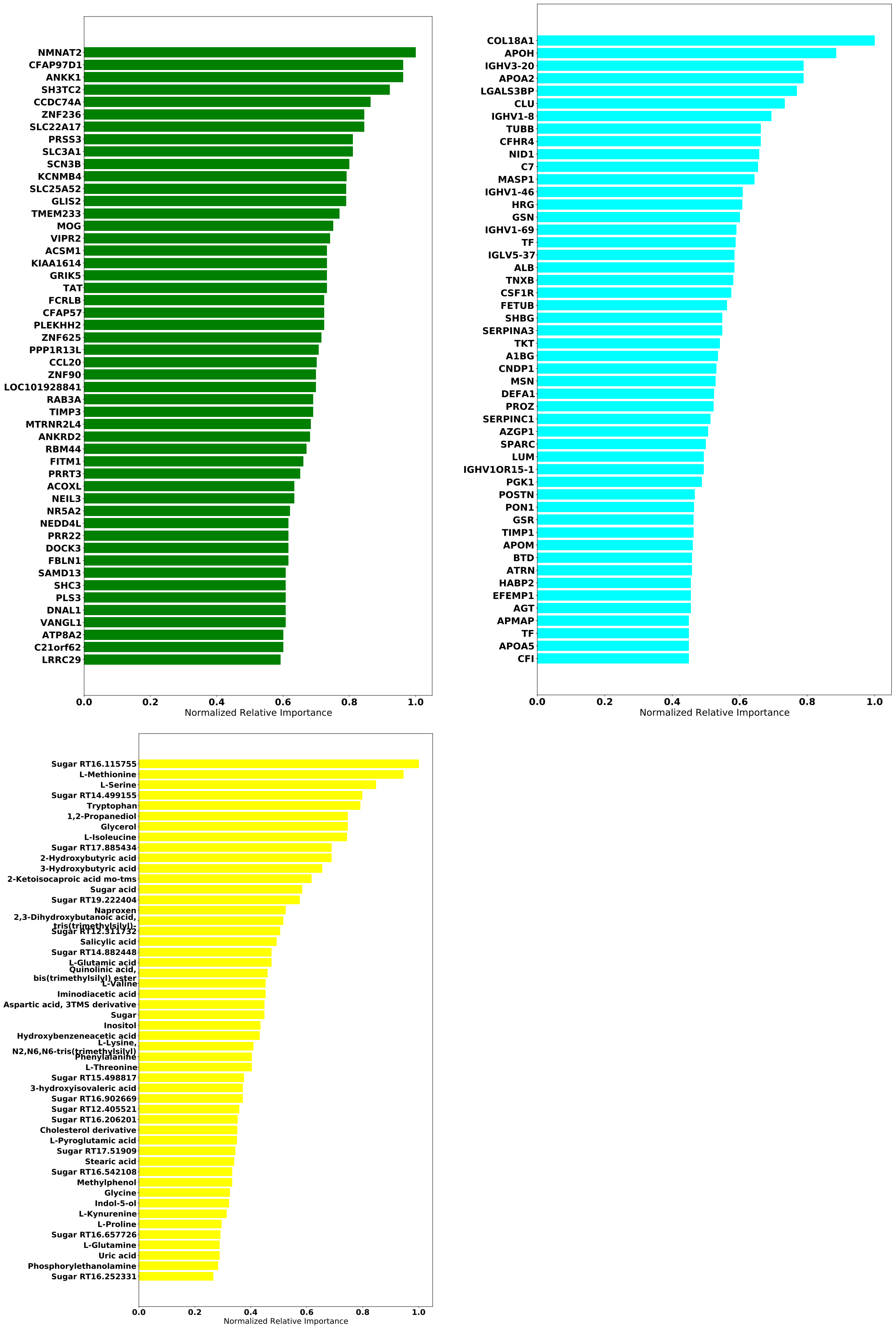

Figure 2: Feature importance plots of the omics data used in the COVID-19 application. Upper left: RNA-Seq; upper right: Proteomics ; lower left: Metabolomics. Feature importance for each variable was normalized to the feature ranked highest for each omics.

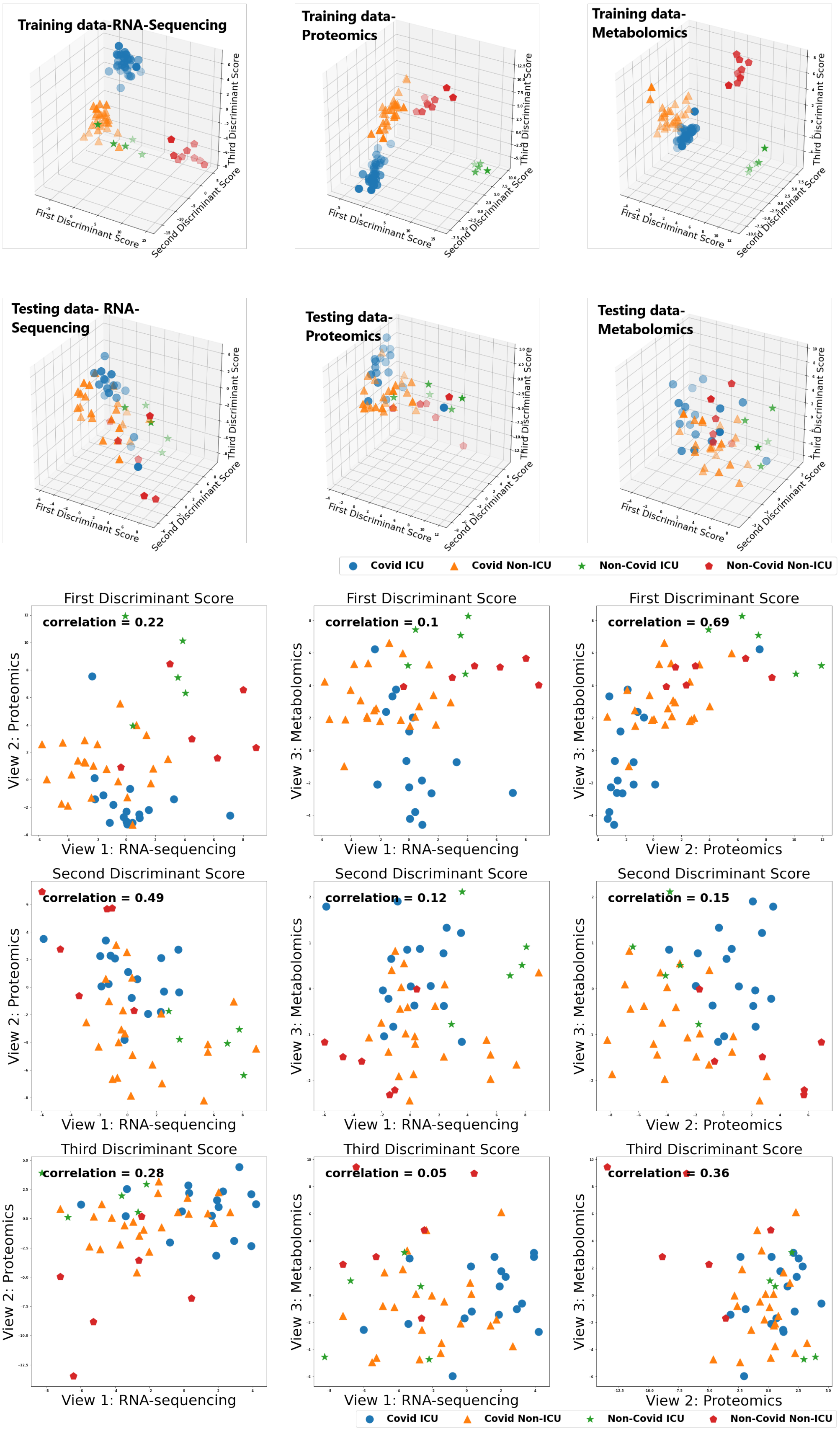

Figure 3: Discrimination (3-D) plots: COVID-19 patient groups are well-separated in the training data. From the testing data, the COVID ICU group seems to be separated from the COVID NON-ICU and NON-COVID ICU groups, especially in the RNA-sequencing and proteomics data. Correlation plots (2-D): Overall (combining all three discriminant scores), the mean correlation between the metabolomics and proteomics data was highest (0.4) while the mean correlation between the metabolomics and RNA-sequencing data was lowest (0.09).

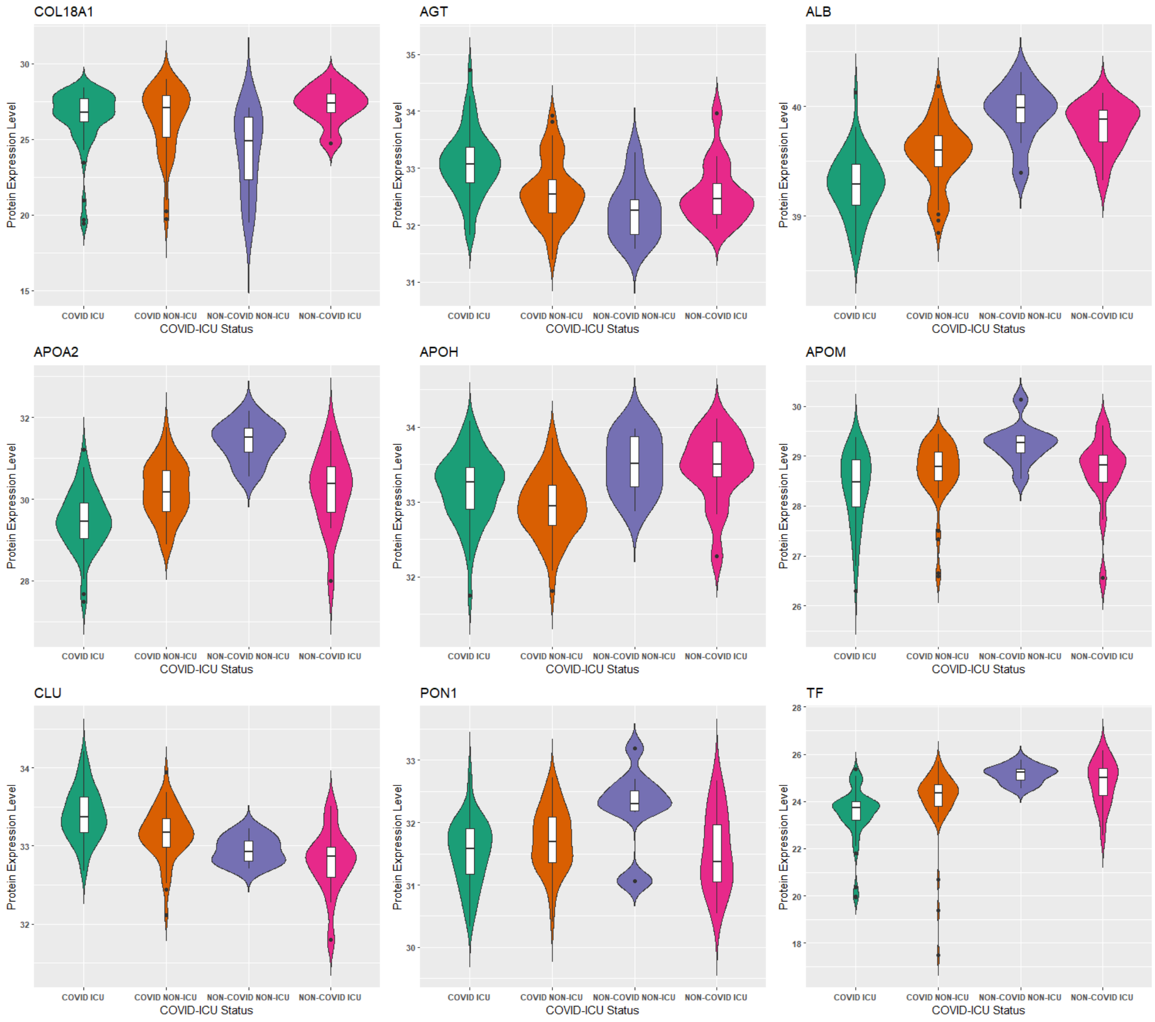

Figure 4: Comparison of protein levels among COVID-19 patient groups (p-value < 0.05, Kruskal-Wallis test). COL18A1 was highly ranked by Deep IDA, and the other 8 proteins are shared by the "FXR/RXR Activation" and "LXR/RXR Activation" pathways. Protein expression levels for ALB, APOM, and TF are lower in patients with COVID-19 (especially in patients with COVID-19 who were admitted to the ICU). Protein expression levels for AGT and CLU are higher in patients with COVID-19 admitted to the ICU compared to the other groups.

Table 4: Top 5 Canonical Pathways from Ingenuity Pathway Analysis (IPA).

**Omics Data**	**Top Canonical Pathway**	**P-value**	**Molecules Selected**
RNA Sequencing	4-hydroxybensoate Biosynthesis	2.07E-03	TAT
	4-hydroxyphneylpyruvate Biosythesis	2.07E-03	TAT
	Tyrosine Degradation 1	1.03E-02	TAT
	Role of IL-17A in Psoriasis	2.86E-02	CCL20
	Fatty Acid Activation	2.86E-02	ACSM1
Proteomics	LXR/RXR Activation	4.14E-11	AGT, ALB, APOA2, APOH, APOM, CLU, PON1, TF
	FXR/RXR Activation	5.02E-11	AGT, ALB, APOA2, APOH, APOM, CLU, PON1, TF
	Acute Phase Response Signaling	3.30E-08	AGT, ALB, APOA2, APOH, HRG, SERPINA3, TF
	Atherosclerosis Signaling	1.06E-07	ALB, APOA2, APOM, CLU, COL18A1, PON1
	Clathrin-mediated Endocytosis Signaling	1.09E-06	ALB, APOA2, APOM, CLU, PON1, TF
Metabolomics	tRNA Charging	2.25E-13	L-glutamic acid, L-Phenylalanine, L-Glutamine, Glycine, L-Serine, L-Methionine; L-Valine, L-Isoleucine, L-Threonine, L-Tryptophan, L-Proline
	Glutamate Receptor Signaling	5.43E-05	L-glutamic acid, glycine, L-Glutamine
	Phenylalanine Degradation IV (Mammalian, via Side Chain)	7.24E-05	L-glutamic acid, glycine, L-Glutamine, L-Phenylalanine
	Superpathway of Serine and Clycine Biosynthesis I	3.28E-04	L-glutamic acid, glycine, L-serine
	y-glutamyl Cycle	4.23E-04	L-glutamic acid, glycine, pyrrolidonecarboxylic acid

Table 5: Top Diseases and Biological Functions from Ingenuity Pathway Analysis (IPA).

	Top Diseases and Bio Functions	P-value range	Molecules Selected
RNA Sequencing	Cancer (such as non-melanoma solid tumor, head and neck tumor)	4.96E-02 – 2.74E-05	48
	Organismal Injury and Abnormalities	4.96E-02 – 2.74E-05	48
	Neurological Disease (such as glioma cancer, brain lesion, neurological deficiency)	4.86E-02 – 5.84E-05	36
	Developmental Disorder (such as intellectual diability with ataxia)	4.46E-02 – 3.76E-05	16
	Hereditary Disorder (such as familial midline effect)	4.86E-02 – 2.02E-05	16
Proteomics	Infectious Diseases (such as Severe COVID-19, COVID-19, infection by SARS coronavirus)	1.75E-03 – 8.31E-13	19
	Inflammatory Response (such as inflammation of organ, degranulation of blood platelets)	1.29E-03 – 1.34E-12	32
	Metabolic Disease (such as amyloidosis, Alzheimer disease, diabetes mellitus)	1.47E-03 – 2.48E-12	20
	Organismal Injury and Abnormalities (such as amyloidosis, tauopathy)	1.72E-03 – 2.48E-12	39
	Neurological Disease (such as tauopathy, progressive encephalopathy, progressive neurological disorder)	1.57E-03 – 3.41E-11	34
Metabolomics	Cancer	3.63E-02 – 5.20E-14	18
	Gastrointestinal Disease (such as digestive system cancer, hepatocellular carcinoma)	3.64E-02 – 5.20E-14	20
	Organismal Injury and Abnormalities (such as digestive system cancer, abdominal cancer)	3.79E-02 – 5.20E-14	22
	Hepatic System Disease (such as hepatocellular carcinoma, liver lesion)	2.91E-02 – 1.66E-11	15
	Developmental Disorder (such as mucopolysaccharidosis type I, spina bifida)	2.44E-02 – 1.83E-09	11

Table 6: Top Molecular and Cellular Functions Functions from Ingenuity Pathway Analysis (IPA).

	Molecular and Cellular Functions	P-value range	Molecules Selected
RNA Sequencing	Cell Death and Survival	4.46E-02 – 2.00E-03	8
	Amino Acid Metabolism	3.47E-02 – 2.07E-05	2
	Cell-to-cell Signaling and Interaction	4.86E-02 – 2.07E-03	10
	Cellular Assembly and Organization	4.46E-02 – 2.07E-03	9
	Cellular Function and Maintenance	4.86E-02 – 2.07E-03	10
Proteomics	Cellular Compromise	1.29E-03 – 1.34E-12	13
	Cellular Movement	1.65E-03 – 2.19E-09	24
	Lipid Metabolism	1.28E-03 – 2.95E-09	15
	Molecular Transport	1.28E-03 – 2.95E-09	15
	Small molecule Biochemistry	1.61E-03 – 2.95E-09	19
Metabolomics	Amino Acid Metabolism	3.64E-02 – 3.99E-08	9
	Molecular Transport	3.82E-02 – 3.99E-08	17
	Small Molecule Biochemistry	3.64E-02 – 3.99E-08	18
	Cellular Growth and Proliferation	3.79E-02 – 5.12E-08	16
	Cell Cycle	3.63E-02 – 5.81E-07	10

Table 7: **Linear Simulations** Network structures for all deep learning based methods. In order to make fair comparisons, for each dataset, the network structure for Deep CCA/Deep GCCA is the same as the proposed Deep IDA method. The activation function is Leakly Relu with parameter 0.1 by default. After activation, batch normalization is also implemented. – indicates not applicable

Data	Sample size (Train,Valid,Test)	Feature size (p^1, p^2, p^3)	Method	Network structure	Epochs per run	Batch size
Setting 1	540,540,1080	1000,1000,-	Deep IDA (+Bi-Bootstrap)	Input-512-256-64-10	50	540
Setting 1	540,540,1080	1000,1000,-	Deep CCA	Input-512-256-64-10	50	180
Setting 2	540,540,1080	1000,1000,1000	Deep IDA (+Bi-Bootstrap)	Input-512-256-20	50	540
Setting 2	540,540,1080	1000,1000,1000	Deep GCCA	Input-512-256-64-20	200	540

Table 8: **Nonlinear Simulations** Network structures for all deep learning based methods. In order to make fair comparisons, for each dataset, the network structure for Deep CCA/Deep GCCA is the same as the proposed Deep IDA method. The activation function is Leakly Relu with parameter 0.1 by default. After activation, batch normalization is also implemented.

Data	Sample size (Train,Valid,Test)	Feature size (p^1, p^2, p^3)	Method	Network structure	Epochs per run	Batch size
Setting 1	350,350,350	500,500	Deep IDA (+Bi-Bootstrap)	Input-256*10-64-20	50	350
Setting 1	350,350,350	500,500	Deep CCA	Input-256*10-64-20	50	350
Setting 2	5250,5250,5250	500,500	Deep IDA (+Bi-Bootstrap)	Input-256-256-256-256-256-256-64-20	50	500
Setting 2	5250,5250,5250	500,500	Deep CCA	Input-256-256-256-256-256-256-64-20	50	500
Setting 3	350,350,350	2000,2000	Deep IDA (+Bi-Bootstrap)	input-256-256-256-256-256-256-256-64-20	50	350
Setting 3	350,350,350	2000,2000	Deep CCA	input-256-256-256-256-256-256-256-64-20	50	350
Setting 4	5250,5250,5250	2000,2000	Deep IDA (+Bi-Bootstrap)	Input-256-256-256-256-256-256-64-20	50	500
Setting 4	5250,5250,5250	2000,2000	Deep CCA	Input-256-256-256-256-256-256-64-20	50	500

Table 9: **Real Data Analysis** Network structures for all deep learning based methods. In order to make fair comparisons, for each dataset, the network structure for Deep CCA/Deep GCCA is the same as the proposed Deep IDA method. The activation function is Leakly Relu with parameter 0.1 by default. After activation, batch normalization is also implemented. For Covid-19 data, we select the top 50 features for each view from Bootstrap Deep IDA with input-512-20. $-$ indicates not applicable

Data	Sample size (Train,Valid,Test)	Feature size (p^1, p^2, p^3)	Method	Network structure	Epochs per run	Batch size
Noisy MNIST	50000,10000, 10000	784,784,-	Deep CCA non-Bootstrap Deep IDA	Input-512-256-64-20	50	50000
Covid-19	74,0,21	2734,269,66	non-Bootstrap Deep IDA	Input-512-20	20	74
Covid-19	74,0,21	2734,269,66	Deep IDA on selected top 50 features	Input-512-256-20	20	74
Covid-19	74,0,21	2734,269,66	Deep IDA on selected top 10 percent features	Input-256-64-20	20	74
Covid-19	74,0,21	2734,269,66	Deep GCCA on selected top 50 features	Input-256-20	150	74

References

[1] Galen Andrew, Raman Arora, Jeff Bilmes, and Karen Livescu. Deep canonical correlation analysis. Journal of Machine Learning Research: Workshop and Conference Proceedings, 2013.

[2] Adrian Benton, Huda Khayrallah, Biman Gujral, Dee Ann Reisinger, Sheng Zhang, and Raman Arora. Deep generalized canonical correlation analysis. Proceedings of the 4th Workshop on Representation Learning for NLP (RepL4NLP-2019), page 1–6, 2019.

[3] Matthias Dorfer, Rainer Kelz, and Gerhard Widmer. Deep linear discriminant analysis. arXiv preprint arXiv:1511.04707, 2015.

[4] Trevor Hastie, Robert Tibshirani, and Jerome Friedman. The Elements of Statistical Learning: Data Mining, Inference, and Prediction (Second Edition). Springer, 2009.

[5] Peng Hu, Dezhong Peng, Yongsheng Sang, and Yong Xiang. Multi-view linear discriminant analysis network. IEEE Transactions on Image Processing, 28(11):5352–5365, 2019.

[6] Diederik P Kingma and Jimmy Ba. Adam: A method for stochastic optimization. arXiv preprint arXiv:1412.6980, 2014.

[7] Yann LeCun, L´eon Bottou, Yoshua Bengio, and Patrick Haffner. Gradient-based learning applied to document recognition. Proc. IEEE, (11):2278–2324, 1998.

[8] Andrew L Maas, Awni Y Hannun, and Andrew Y Ng. Rectifier nonlinearities improve neural network acoustic models. ICML, 2013.

[9] Ali Mirzaei, Vahid Pourahmadi, Mehran Soltani, and Hamid Sheikhzadeh. Deep feature selection using a teacher-student network. arXiv:1903.07045, 2019.

[10] Sehwan Moon and Hyunju Lee. MOMA: a multi-task attention learning algorithm for multi-omics data interpretation and classification. Bioinformatics, 38(8):2287–2296, 02 2022.

[11] Adam Paszke, Sam Gross, Francisco Massa, Adam Lerer, James Bradbury, Gregory Chanan, Trevor Killeen, Zeming Lin, Natalia Gimelshein, Luca Antiga, Alban Desmaison, Andreas Kopf, Edward Yang, Zachary DeVito, Martin Raison, Alykhan Tejani, Sasank Chilamkurthy, Benoit Steiner, Lu Fang, Junjie Bai, and Soumith Chintala. Pytorch: An imperative style, high-performance deep learning library. In Advances in Neural Information Processing Systems 32, pages 8024–8035. Curran Associates, Inc., 2019.

[12] Sandra E. Safo, Eun Jeong Min, and Lillian Haine. Sparse linear discriminant analysis for multiview structured data. Biometrics, 2021.

[13] Weiran Wang, Raman Arora, Karen Livescu, and Jeff Bilmes. On deep multi-view representation learning. Journal of Machine Learning Research: Workshop and Conference, 2015.
